# Balance Adaptation While Standing on a Compliant Base Depends on the Current Sensory Condition in Healthy Young Adults

**DOI:** 10.3389/fnhum.2022.839799

**Published:** 2022-03-25

**Authors:** Stefania Sozzi, Marco Schieppati

**Affiliations:** ^1^Centro Studi Attività Motorie (CSAM), Istituti Clinici Scientifici Maugeri SB (IRCCS), Pavia, Italy; ^2^Istituti Clinici Scientifici Maugeri SB (IRCCS), Pavia, Italy

**Keywords:** balance, adaptation, stance, repeated trials, sensory conditions, compliant surface, vision, touch

## Abstract

**Background:**

Several investigations have addressed the process of balance adaptation to external perturbations. The adaptation during unperturbed stance has received little attention. Further, whether the current sensory conditions affect the adaptation rate has not been established. We have addressed the role of vision and haptic feedback on adaptation while standing on foam.

**Methods:**

In 22 young subjects, the analysis of geometric (path length and sway area) and spectral variables (median frequency and mean level of both total spectrum and selected frequency windows) of the oscillation of the centre of feet pressure (CoP) identified the effects of vision, light-touch (LT) or both in the anteroposterior (AP) and mediolateral (ML) direction over 8 consecutive 90 s standing trials.

**Results:**

Adaptation was obvious without vision (eyes closed; EC) and tenuous with vision (eyes open; EO). With trial repetition, path length and median frequency diminished with EC (*p* < 0.001) while sway area and mean level of the spectrum increased (*p* < 0.001). The low- and high-frequency range of the spectrum increased and decreased in AP and ML directions, respectively. Touch compared to no-touch enhanced the rate of increase of the low-frequency power (*p* < 0.05). Spectral differences in distinct sensory conditions persisted after adaptation.

**Conclusion:**

Balance adaptation occurs during standing on foam. Adaptation leads to a progressive increase in the amplitude of the lowest frequencies of the spectrum and a concurrent decrease in the high-frequency range. Within this common behaviour, touch adds to its stabilising action a modest effect on the adaptation rate. Stabilisation is improved by favouring slow oscillations at the expense of sway minimisation. These findings are preliminary to investigations of balance problems in persons with sensory deficits, ageing, and peripheral or central nervous lesion.

## Introduction

Standing quietly may not require a particular neural effort (Morasso and Schieppati, 1999; Ivanenko and Gurfinkel, 2018) such that even a cognitive task is performed better while standing than sitting in young healthy subjects (Rosenbaum et al., 2017). Understandably, the metabolic cost of quiet stance is low in healthy subjects unless they have to counteract perturbations (Houdijk et al., 2009). However, standing in critical conditions, such as in tandem stance or on foam, is an attention-demanding task, where the cognitive task may affect the level of instability (Boisgontier et al., 2013; Honeine et al., 2017). Further, maintaining the equilibrium in critical conditions requires continuous activity in several muscles (Schieppati et al., 1995; Tokuno et al., 2007; Kelly et al., 2012; Sozzi et al., 2013), implying a considerable effort. When standing on foam, muscle activity and metabolic cost increases (Fransson et al., 2007; Mohapatra et al., 2014; Mademli et al., 2021) and body sway increases concurrently (Teasdale et al., 1991; Patel et al., 2011; Anson et al., 2019; Hsiao et al., 2020).

Keeping the body vertical, the ultimate goal of the control of upright stance (Hlavačka et al., 1996; Jahn and Wühr, 2020) is achieved thanks to continuous firing of the receptors activated by gravity, movement of body segments and centre of mass, and by changes in the relation between foot sole and basis of support (Mittelstaedt, 1998; see Felicetti et al., 2021). An inventory of all responsible receptors is implausible. One can only hope to itemise most of those for which reports are available, based on experiments conducted in quiet and perturbed stance conditions (Borel et al., 2008; Creath et al., 2008; Pettorossi and Schieppati, 2014; Cheung and Schmuckler, 2021; Nedelkou et al., 2021). In the same vein, it is virtually impossible to indicate all the muscles more or less active when standing on a compliant surface or under similarly critical postures, such as standing in tandem or on one leg. Standing on foam requires continuous adjustments of all body segments, aiming at achieving core stability by appropriate recruitment of muscle activity (Grüneberg et al., 2005; Gurfinkel et al., 2006; Sozzi et al., 2013; Küng et al., 2009). Proprioceptive inflow must be strongly enhanced when standing on foam, including the input from the intrinsic foot muscles (Schieppati et al., 1995; Abbruzzese et al., 1996; Forestier et al., 2015). A proprioception-based mechanism is at the origin of implicit learning and adaptation (Fitzpatrick et al., 1992; Rosenkranz and Rothwell, 2012; see, in a different context, Tsay et al., 2021) and favours the synergistic activity of the postural muscles (Danna-Dos-Santos et al., 2008).

We posited that a large amount of sensory barrage during standing on foam (Jeka et al., 2004), the unfamiliar and atypical activation of foot sole skin and intrinsic muscle receptors (Duysens et al., 2008), and the abundance in muscle activation (see Latash, 2012) would be conditions favouring sensory channel reweighting and adaptation over time of body sway with task repetition, ideally leading to enhanced body stability (Lhomond et al., 2021). Therefore, we explored the possibility that balance control adaptation would actually occur, and investigated the process of adaptation under different sensory conditions during the repetition of standing on-foam trials, as a development of a recently published paper on the specific effects of distinct sensory conditions on standing balance (Sozzi et al., 2021). Different sensory channels would be more or less sensitive to standing on foam, and the complex task of maintaining the equilibrium would be assisted by appropriately modulating the weight of the information from these channels (Mahboobin et al., 2009).

Adaptation has received much attention when repeated perturbations of balance have been investigated. The responses to a consecutive set of perturbation trials become usually smaller than those to the initial trials. Perturbations of balance can have various configurations, like translations or tilts of the base of support (Gollhofer et al., 1989; Corna et al., 1999; Schmid et al., 2011; Welch and Ting, 2014; Sozzi et al., 2020) or sudden changes in the velocity of the treadmill upon which subjects walk (Patel and Bhatt, 2015; McCrum et al., 2017) or else strikes of a loaded pendulum hitting the upper body of a standing person (Kaewmanee et al., 2020). Also, translational visual stimuli challenge the sensory reweighting mechanisms (Isableu et al., 2011; Fransson et al., 2019), much as occurs for vestibular stimulation (St George and Fitzpatrick, 2011; Barmack and Pettorossi, 2021) or muscle vibration (Bove et al., 2006; Duysens et al., 2008).

Under all settings, modulation of balancing behaviour appears in various forms and time scales. When balancing on a platform translating in AP direction, virtually no adaptation with vision takes place, and the steady-state of body segment motion and muscle activity is reached immediately after the startle reaction (Oude Nijhuis et al., 2010). This is explained by the strong stabilising effect of vision during responses to support surface translation (Akçay et al., 2021). Conversely, occluding vision produces a prolonged adaptation process, whereby the values of mechanical and EMG variables decay over time and reflex responses to muscle stretch diminish at a higher rate than the anticipatory activities (Sozzi et al., 2016). The weight of vision increases under challenging conditions (Loughlin et al., 1996; Patel et al., 2011; Lee et al., 2018) and when proprioception is disturbed (Isableu et al., 2011; Goodworth et al., 2014).

During postural perturbations and during walking, body stability is enhanced by light touch to a fixed frame (Dickstein and Laufer, 2004; Forero and Misiaszek, 2013; Martinelli et al., 2015; Kaulmann et al., 2020). Light touch rapidly reaches the somatosensory cortex (Schieppati and Ducati, 1984) and deactivation of the sensory cortex is related to worse balance performance in old age (Noohi et al., 2019). Haptic information, be it by tactile passive sensory inputs or light fingertip touch, has an effect comparable to vision (Holden et al., 1994; Rogers et al., 2001; Honeine and Schieppati, 2014; Chen et al., 2018) or to the vestibular system (Creath et al., 2008), even when standing on foam (Krishnan and Aruin, 2011; Albertsen et al., 2012). The continuous haptic input from the fingertip lightly touching an earth-fixed surface improves the control of upright stance under both stable or unstable stance (Clapp and Wing, 1999), after a perturbation (Johannsen et al., 2007) and during walking (Dickstein and Laufer, 2004; Forero and Misiaszek, 2013; Martinelli et al., 2015; Kaulmann et al., 2020) and in patients with poor balance (see Horak, 2009; Baldan et al., 2014). Hence, vision, proprioception, and haptic feedback have substantial effects in helping the body cope with perturbations of upright stance.

In the present study, we have addressed in particular the role of vision and haptic sense in the process of adaptation of stance on a compliant surface, in the absence of any external perturbation (Singh et al., 2012; Sozzi et al., 2012). We have hypothesised that adaptation would occur during repeated and prolonged standing-on-foam trials, that stance control would progressively focus on and rely on the sensory input available, and that vision and touch would differently affect adaptation. We asked the following questions. Does adaptation depend on the type (or number) of the available sensory information? Does adaptation modify the quality of stance by attaining a “default” oscillation mode, equal for distinct sensory condition(s)? Would the adapted state witness the nature of the postural control mode across specific sensory conditions? Does adaptation under light-touch conditions depend on progressive modifications of the level of force exerted by the finger? Do excursions in the frontal and sagittal plane undergo similar or different changes with adaptation, and if so is this contingent on the sensory condition?

Instead of focussing the analysis on the conventional metrics of sway, such as area and length of excursions of the centre of feet pressure (CoP), we asked whether particular frequencies of body oscillations would show specific changes during and at the end of the adaptation period, depending on the sensory information available. Hence, we leveraged the frequency analysis of the CoP excursions, based on the recent demonstration of the differences in the pattern of body stabilisation in the presence of vision compared to haptic sense (Sozzi et al., 2021). Here, we hypothesised that these inputs, yielding information of a different nature about the environment and probably processed through different brain pathways, also affect in a partially different way the process of adaptation.

## Materials and Methods

### Participants

In this study, 22 healthy young adults (10 men and 12 women) participated. Age was 28.6 ± 4.4 years (mean ± *SD*), height 172.1 ± 7.1 cm and weight 67.5 ± 13.2 kg. The participants were resident physicians or physiotherapists at the Istituti Clinici Scientifici Maugeri SB. All were in good conditions, had no sight problems, or their visual acuity was corrected, were free of neurological and musculo-skeletal disorders, and none had had vertigo episodes in the past. No participant reported injuries or occurrences of falls in the previous year. All provided written, informed consent to participate in the experiment as conformed to the Declaration of Helsinki. The local review board approved the research protocol (Istituti Clinici Scientifici Maugeri SB, approval number #2564-CE). The size of the target population was based on previous experience and on the convenience of collecting the data. Since prior information is lacking, the power of the applied statistical test was assessed once the sample was collected.

### Procedures

Subjects stood barefoot, for at least 100 s on a force platform (Kistler 9286BA, Switzerland) on which a foam pad (Airex Balance Pad, Sins, Switzerland, L 50 cm × W 41 cm × H 6 cm, density 55 g/dm^3^, Young’s module 260 kP) was placed (Lin et al., 2015). The outer profiles of the parallel feet were set at hip-width. Balance was measured under four standing conditions: with eyes open (EO), with eyes closed in the absence of touch (EC), and with light-touch without (EC-LT) or with vision (EO-LT). The position of the feet was marked on a paper sheet placed on top of the foam pad for consistency across consecutive trials. Subjects were asked to stand at ease (Hufschmidt et al., 1980) and to look at the visual scene of the laboratory wall at a 6 m distance (Sozzi et al., 2021). All subjects were naive to foam-standing. They were asked to avoid voluntary head movements in pitch, roll, and yaw, and to minimally move the eyes during the EO trials. In the EC conditions (EC and EC-LT), subjects closed their eyes before the start of the acquisition and kept their eyes closed until the end of the acquisition epoch. In the touch conditions (EC-LT and EO-LT), the instruction was to maintain a constant light touch with the index finger on the surface of the haptic device. They spontaneously chose their hand for the finger-touch task. At the end of the first trial, we asked whether that hand was the hand they used most frequently for the daily activities. The answer was affirmative in all cases and the hand was the right one. The haptic device consisted of a flat horizontal wooden square (10 cm × 10 cm) fixed on top of a strain gauge, located at about the height of the belly button and distant about 15 cm from it in the sagittal plane. When the force applied to the touchpad exceeded 1 N, the device beeped and subjects adjusted the force applied with the fingertip. This occurred rarely, mostly in the initial time period before the start of the acquisition. There was no instruction to keep the finger immobile on the force pad, so that small fluctuations in the hand and finger position were allowed. The finger never slipped off the force pad. During the no-touch conditions, both with eyes closed and eyes open, participants kept their arms relaxed by their sides. Intervals between trials were 15–30 s long when the subject stepped off the force platform and made a few steps. Before starting each consecutive trial, the experimenter verified the foot’s position on the foam pad.

Each volunteer came to the laboratory four times, on separate days. Each day subjects completed eight equal-duration (∼100 s) consecutive standing trials in one of the sensory conditions of interest (EC, EO, EC-LT, and EO-LT). The order of the sensory conditions was randomised across subjects. There was no preliminary practice trial. The last 90 s-epoch of each 100 s-stance trial was acquired, to exclude the adjusting phase on stepping onto the foam pad. None of the subjects lost balance while standing on foam despite the increase in sway compared to a solid base of support (Sozzi et al., 2021). When asked at the end of the trial sequence, subjects reported no fatigue.

### Data Acquisition and Processing

Details about data acquisition, processing, and identification of the frequency windows of interest in the spectrum are reported in Sozzi et al. (2021). Briefly, the platform data, from which the CoP was calculated, and the force data from the haptic device onto which the fingertip was resting, were acquired at the sampling frequency of 140 Hz by dedicated software (Smart-D, BTS, Italy). *Post hoc* analysis was done using Excel (Microsoft), MATLAB (Mathworks), and LabVIEW (National Instrument). The force platform signals of the CoP excursions along with the anteroposterior (AP) and mediolateral (ML) directions were high-pass filtered at 0.01 Hz and low-pass filtered at 20 Hz with a 4th order Butterworth filter, after removing the respective mean values. The length of the sway path was the total length of the wandering CoP, and the sway area was the surface of the 95% ellipse fitted to the dispersion of the time-series of the AP plotted against the corresponding ML data (Schubert and Kirchner, 2014).

The frequency analysis was performed by the fast Fourier transform of the CoP ML and AP time-series of each trial, subject, and sensory condition, employing the Auto power spectrum VI algorithms of the LabVIEW functions. The duration of the acquired epoch and the sampling rate defined the lowest and the highest detectable frequency, respectively (Michalak and Jakowski, 2002; Sozzi et al., 2021). The resolution (sample frequency/sample number) was 0.011 Hz for a sampling frequency of 140 Hz and a sample number of 12,600 (equal to 90*140). The power spectrum signal was expressed in cm^2^_*rms*_. For each subject, trial, and sensory condition, we calculated the median frequency (that divides the power spectrum into two parts of equal area) and the mean level of the spectrum (the arithmetic mean of the amplitude values at each sampled frequency), which represents an index of the oscillation amplitude (Krizková et al., 1993; Nagy et al., 2004). These variables were calculated between 0.01 and 2 Hz and for both AP and ML directions. Distinct frequency windows (W) were identified to investigate in some detail the effect of adaptation on the frequency content of the spectrum of the ML and AP excursions of the CoP. The segmentation was based on the power spectrum profile of the EC condition, which featured the greatest overall amplitude compared to all other conditions. The procedure was based on the study of Sozzi et al. (2021) and consisted in locating the boundaries of the windows by detecting the local minima of the profile of the mean power spectrum and its standard deviation in successive epochs of 0.05 Hz. Six minima were thus defined (Sozzi et al., 2021). The same minima were used for the window definition in both ML and AP directions. Hence, ranges were: W1, 0.01–0.055 Hz; W2, 0.056–0.2 Hz; W3, 0.21–0.43 Hz; W4, 0.44–0.8 Hz; W5, 0.81–1.31 Hz; and W6, 1.32–2 Hz.

### Data Treatment and Statistics

Centre of feet pressure (CoP) path length and sway area, median spectrum frequency, the mean level of the full spectrum (from 0.01 to 2 Hz) and of the distinct frequency windows were used to assess the time course of the changes in these variables over the eight consecutive repetitions. We postulated that an exponential model would fit the trends, as described on several other occasions (Tjernström et al., 2010; Schmid et al., 2011; Welch and Ting, 2014; Patel and Bhatt, 2015; Sozzi et al., 2020). To compare these trends across conditions, all data were log-transformed, and regression lines fitted to these data, even if for certain participants, sensory conditions, metrics, and frequency windows an exponential trend was not obvious. The slope of the regression lines was considered an index of the adaptation rate. The slopes of the lines obtained for each subject were averaged in each sensory condition and compared with zero (no adaptation) by the Student’s *t*-test. In addition, separately for each sensory condition, the mean level of the spectrum in each window for the trials from 2 to 8 was expressed as a percent of the mean level of the spectrum of the first trial and graphed in a radar plot.

Assumptions for parametric statistics, as assessed by the Kolmogorov-Smirnov test and Levene’s test, were met for all variables of interest. The effects of the different sensory conditions on path length and sway area of the CoP were compared by 4 (sensory conditions) × 8 (trial repetition) repeated measure (rm) ANOVA. The slopes of the regression lines fitted on path length and sway area were compared by a 1-way rm ANOVA. A 4 (sensory conditions) × 8 (trial repetition) rm ANOVA was used to compare the median frequency and the mean level of the spectrum between 0.01 and 2 Hz, separately for the ML and AP directions. The slope of the regression lines fitted to the median frequency and the mean level of the spectrum were compared by a 2 (ML and AP directions) × 4 (sensory conditions) rm ANOVA. A 2 (ML and AP directions) × 4 (sensory conditions) × 6 (frequency windows) rm ANOVA was used to compare the slope of the regression lines fitted to the mean level of the spectrum in each frequency window. A 2 (EC-LT and EO-LT conditions) × 8 (trial repetition) rm ANOVA was used to compare the force exerted by the subjects on the touchpad between EC-LT and EO-LT conditions. Where the differences were significant, the η^2^*_*p*_* was reported. The minimum effect size given our sample size (*n* = 22) was calculated using G*Power (Faul et al., 2007). Given our sample, the study proved to have a sufficient power (>80%) to detect an effect size *d* of 0.55. The *post hoc* test was Fisher’s LSD test. The significance level was set at 0.05. Statistical tests were performed using Statistica (Statsoft, United States).

## Results

**FIGURE 1:**
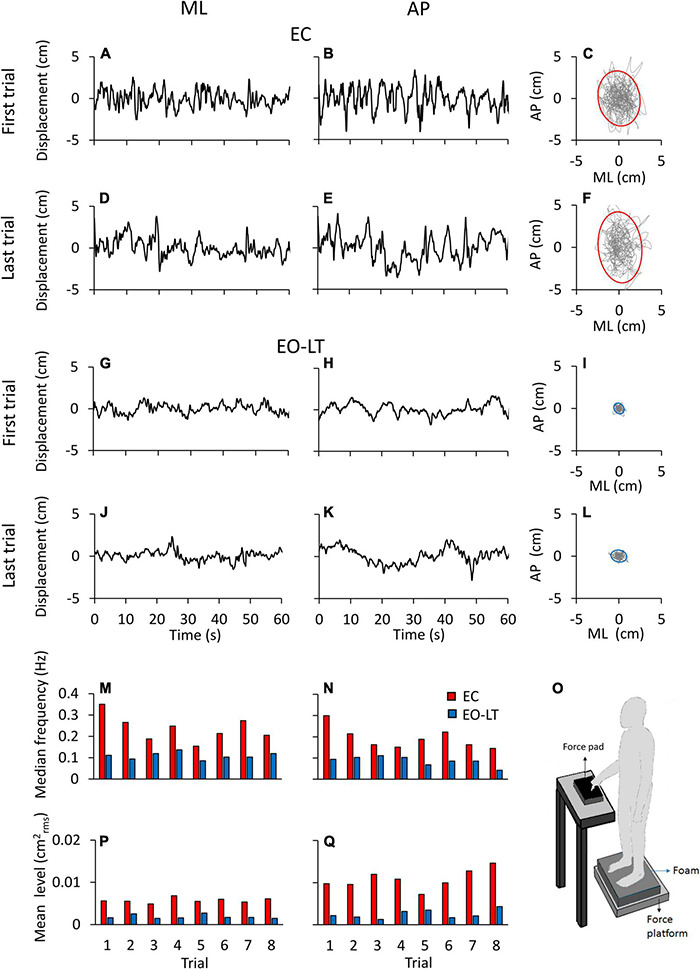
Centre of feet pressure (CoP) excursions and effects of trial repetition on the power spectrum in a representative subject. The recorded CoP displacement in the ML **(A,D,G,J)** and AP **(B,E,H,K)** directions are reported for the first and last trials in eyes closed in the absence of touch (EC) **(A–E)** and eyes open with light-touch (EO-LT) **(G–K)** conditions. The diagrams of the CoP sway trajectory (grey line) and the 95% ellipse profile (red for EC and blue for EO-LT) are reported in **(C,F,I,L)**. **(M–Q)** Show the median frequency and the mean level of the spectrum computed for the 8 trials performed in the EC and EO-LT conditions. In **(O)**, a sketch of the experimental condition is shown.

Figure 1 shows examples of the recorded time series (a segment of 60 s is depicted) in one representative subject for two different sensory conditions, namely EC and EO-LT. Figures 1A–L show the CoP traces recorded from the force platform in the ML (left column) and AP (middle column) directions. The recordings of the first and the last (adapted) trial are shown for each sensory condition. In the rightmost panels, the excursion of the CoP in the horizontal plane is reported. The sketch in the bottom right shows the experimental condition. CoP excursions in both ML and AP directions and sway areas were much reduced with vision and touch compared to the EC condition. The two bottom rows show the median frequency values and the mean level of the spectrum for each of all eight trials of the same subject and conditions above. The median frequencies diminished with trial repetition. The level of the spectrum was roughly constant in the ML and increased in the AP direction with trial repetition.

### Effects of the Trial Repetition on Path Length and Sway Area of the Excursions of the Centre of Feet Pressure

**FIGURE 2:**
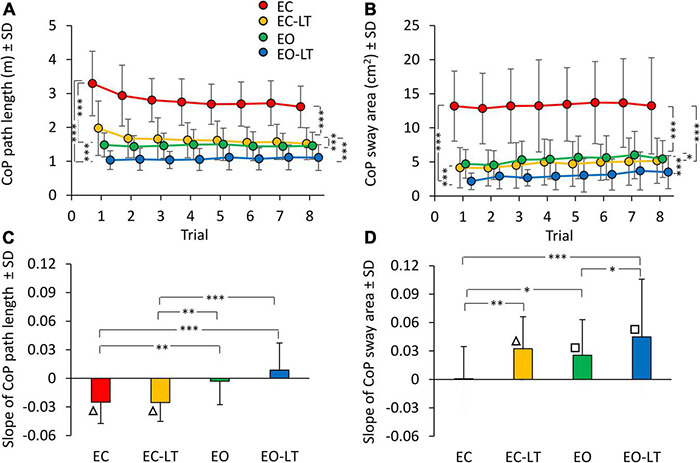
Effect of trial repetition on path length and sway area of CoP excursions under different sensory conditions. Path length **(A)** diminished with trial repetition with EC (red) and EC with light-touch (EC-LT) (yellow), while no adaptation was evident with vision (eyes open (EO), green, and EO-LT, blue). Sway area **(B)** moderately increased with trial repetition for all conditions (less so for EC). **(C,D)** Show the mean slope of the lines fitted to path length and sway area values across the trials. The negative slopes with EC and EC-LT indicate a decrease in path length. Slopes were not different from zero with EO and EO-LT. The slopes for the sway area were positive, indicating an increase, except for EC. Asterisks indicate significant differences between sensory conditions (*p < 0.05; **p < 0.01; ***p < 0.001). Symbols indicate slopes different from zero (□*p* < 0.01; △*p* < 0.001).

The mean path length of the CoP trace (Figure 2A) during the first trial was clearly longer in EC condition (red), and declined in the order EC > EC-LT > EO > EO-LT. The path lengths in EC and EC-LT (yellow) conditions diminished with trial repetition. Path lengths in EO (green) and EO-LT (blue) conditions faced no progressive decrease over the trials. ANOVA main effect showed a difference across sensory conditions [*F*_(3, 63)_ = 151.6, *p* < 0.0001, η^2^*_*p*_* = 0.88]. The *post hoc* test found a difference for all paired comparison between sensory conditions (*p* < 0.001). ANOVA showed also a main effect of trial repetition [*F*_(7, 147)_ = 12.1, *p* < 0.0001, η^2^*_*p*_* = 0.37] and an interaction between sensory conditions and trial repetition [*F*_(21, 441)_ = 7.4, *p* < 0.0001, η^2^*_*p*_* = 0.26]. At the end of trial repetition, CoP path length was still the greatest in EC condition (*post hoc*, *p* < 0.001, for all comparisons) and the smallest in EO-LT (*p* < 0.001, for all comparisons). With touch (EC-LT), the path length decreased and became similar to that in the EO condition (*p* = 0.32).

There was an unspecific correspondence between path length and sway area (both were large with EC and small with EO-LT). However, the sway area (Figure 2B) did not follow the same adaptation pattern of path length, because the sway area increased with trial repetition. There was a difference across sensory conditions [*F*_(3, 63)_ = 98.8, *p* < 0.001, η^2^*_*p*_* = 0.82]. The *post hoc* test found a difference between sensory conditions as well (*p* < 0.05, for all comparisons). ANOVA showed a difference among trial repetitions [*F*_(7, 147)_ = 12.1, *p* < 0.01, η^2^*_*p*_* = 0.14]. The interaction between conditions and trial repetition [*F*_(21, 441)_ = 0.5, *p* = 0.95] was not significant. Supplementary Tables 1, 2 show the probability of all the *post hoc* paired comparisons across repetitions for both path length and sway area, respectively. The path length of the first trial was different from that of all the others in EC and EC-LT conditions, and there was a progressive reduction in the mean path length (to about 75 and 80% of their initial value, respectively) that almost reached a floor at about the 4th–5th trial (*p* < 0.001 for all comparisons). In EO and EO-LT, there was no difference between the first trial and that of all the other trials (*p* > 0.19, for all comparisons). Sway area of the first and last trials were not different in EC (*p* = 0.92) and EO (*p* = 0.11) conditions, while a difference between these trials was observed in EC-LT (*p* < 0.05) and EO-LT (*p* < 0.01) conditions. At the end of trial repetition, the sway area was the greatest under EC condition (*post hoc*, *p* < 0.001, for all comparisons) and the smallest under EO-LT (*p* < 0.001, for all comparisons). There was no difference in sway area between EC-LT and EO conditions (*p* = 0.58).

The bottom panels of Figure 2 summarise the effects of the repetitions on CoP path length (Figure 2C) and sway area (Figure 2D) across sensory conditions, as assessed by the adaptation rate. For each subject, a regression line was drawn through the log-transformed values of the successive trials under each of the four sensory conditions. Then, the slopes of the regression lines were averaged. ANOVA showed a difference in the mean slope of path length between sensory conditions [*F*_(3, 63)_ = 11.6, *p* < 0.001, η^2^*_*p*_* = 0.36]. These slopes were different without vision (EC and EC-LT) from those with vision (EO and EO-LT) (*post hoc*, *p* < 0.01, for all comparisons). Conversely, there was no difference in slope between EC and EC-LT (*p* = 0.99) and between EO and EO-LT (*p* = 0.1). Figure 2C shows that the slope of the lines for EC and EC-LT conditions pointed downward (triangles indicate a significant difference from zero, *p* < 0.001). Hence, path length progressively decreased in these conditions, while a flat line fitted EO and EO-LT conditions, implying no adaptation. On the contrary, sway area (Figure 2D) showed no significant progressive reduction for the EC condition, and a moderate but definitely increasing trend (positive slope) for EC-LT, EO, and EO-LT conditions. The slope of sway area was different between sensory conditions [*F*_(3, 63)_ = 6.7, *p* < 0.001, η^2^*_*p*_* = 0.24]. With EC, the slope was the smallest compared to the other sensory conditions (*post hoc*, *p* < 0.05 for all comparisons). There was a slight difference in slope between EO and EO-LT (*p* < 0.05).

### Effect of Trial Repetition on the Mean Level of the Power Spectrum of the Centre of Feet Pressure Excursions in the Distinct Sensory Conditions

**FIGURE 3:**
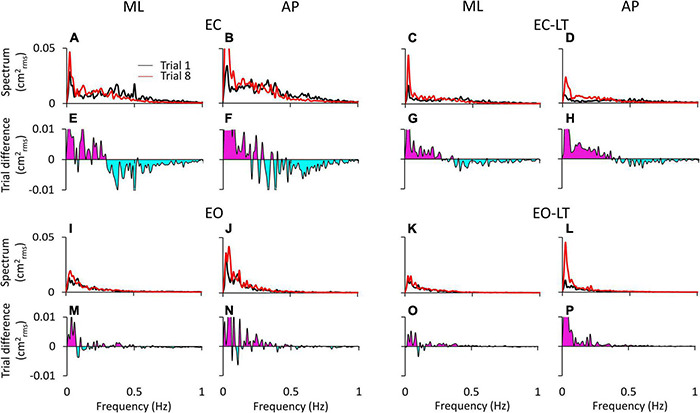
Power spectrum differences between the first and last trials. The profiles of the mean power spectrum of the first (black traces) and last (red traces) trials for both ML and AP directions are superimposed for EC **(A,B)**, EC-LT **(C,D)**, EO **(I,J)**, and EO-LT **(K,L)**. In the panels from **(E–H**,**M**–**P)**, the differences between the spectrum of the last and the first trial (trial 8 minus trial 1) are reported. The pink area indicates a positive difference, i.e., in the corresponding frequency range, the spectrum of the last trial is greater than that of the first trial. The negative differences are highlighted in light blue, indicating that the spectrum of the last trial is smaller than that of the first trial.

Figure 3 shows the difference in the spectrum amplitudes of the last and the first trial for each of the four sensory conditions and the difference between them. The upper traces of each of the four conditions (Figures 3A–D,I–L) show the average profiles of the power spectra of all the subjects. For each panel, the “adapted” profile of the power spectrum (the last trial in the series of eight, red trace) is superimposed to that of the first trial (black trace). All traces pertinent to the ML and AP directions are presented next to each other. The lower traces of each panel are the difference between the spectrum profiles (trial 8 minus trial 1; when the trace moves to the negative part of the graph (light blue area), the amplitude of the last is smaller than that of the first trial, and vice versa for the pink area). Note that in Figure 3, the abscissa has been limited to 1 Hz and the ordinate to 0.05 cm^2^_*rms*_ for a better definition of the result in the lower frequency band.

Overall, the general pattern in the sagittal and frontal planes was roughly similar. Adaptation consistently increased the spectra in the last (red trace) compared to the first trial (black trace) in the low and very-low-frequency range (<0.2–0.3 Hz, pink areas in Figures 3E–H,M–P) in all conditions. These differences were larger without (EC and EC-LT) than with vision (EO and EO-LT) and larger in the AP than ML direction. Conversely, the spectrum mean levels diminished with adaptation in the higher frequency range (from 0.2–0.3 to 1 Hz, light blue areas) particularly with EC, whereas with vision (EO and EO-LT) all the spectra were small and the higher frequencies less represented. With the addition of both vision and touch (EO-LT), subjects felt quite stable from the beginning, as if the stabilising effects of vision and touch added up in assisting the control of stance. Nonetheless, the comparison between the mean profiles of the last and of the first trial featured an isolated, large increase in the lowermost frequencies (from about 0.01 to about 0.1 Hz), larger in AP. The frequencies around 0.2–0.4 Hz, i.e., where the traces of the differences cross the abscissa, did not apparently change between the first and the last trial (A to D).

### Effect of Trial Repetition on the Median Frequency of the Spectrum of the Centre of Feet Pressure Excursions in the Different Sensory Conditions

**FIGURE 4:**
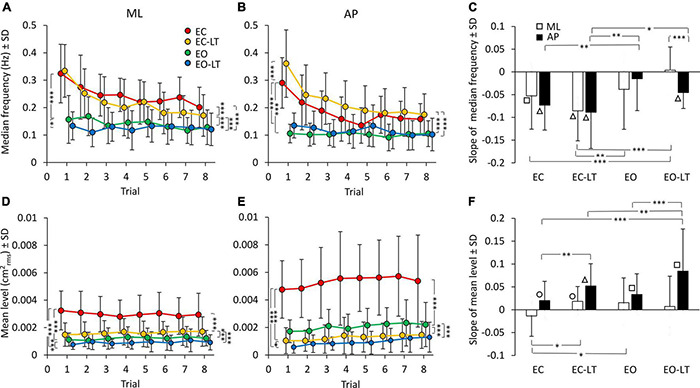
Effect of trial repetition on the median frequency and mean level of the spectrum under the different sensory conditions. Median frequency decreased with trial repetition in the no-vision conditions for both ML **(A)** and AP **(B)** directions. Median frequency was higher in EC (red) and EC-LT (yellow) than in EO (green) and EO-LT (blue) conditions. **(C)** Shows the mean slope of the lines fitted to the median frequency data. Except for EO-LT in the ML direction, all the slopes were negative, indicating a reduction in the value of the median frequency. The mean level of the spectrum for the ML **(D)** and AP **(E)** direction is also reported. For the ML direction, the mean level of the spectrum showed no clear changes with trial repetition, while in the AP direction the mean level of the spectrum increased with trial repetition. **(F)** Shows the mean slope of the lines fitted to the mean levels of the spectrum. Except for the EC in ML direction, all the slopes were positive, indicating an increase of the mean level of the spectrum with the trial repetition. Asterisks indicate significant differences between sensory conditions (*p < 0.05; **p < 0.01; ***p < 0.001). Symbols indicate slopes significantly different from zero (◯*p* < 0.05; □*p* < 0.01; △*p* < 0.001).

The median frequency of the entire power spectrum (0.01–2 Hz) of the CoP excursions of the first trial showed distinct values for each sensory condition. Differences were present for the ML and AP directions as well. The mean values of the median frequency diminished with adaptation in the ML and in the AP direction (Figures 4A,B). The adaptation pattern of the median frequency was similar for EC and EC-LT, as if the absence of vision, regardless of touch, was the main cause of the shift toward low frequencies with trial repetition. Conversely, with vision (EO and EO-LT), the median frequency of the spectra showed little changes over time and was overall smaller (approximately 0.1–0.15 Hz) than that observed for the EC and EC-LT conditions.

ANOVA showed a difference in the median frequency between sensory conditions for ML [*F*_(3, 63)_ = 58.7, *p* < 0.0001, η^2^*_*p*_* = 0.74] and AP directions [*F*_(3, 63)_ = 61.05, *p* < 0.0001, η^2^*_*p*_* = 0.74]. The median frequency was definitely higher with EC and EC-LT than with vision (EO and EO-LT), both in ML (*post hoc*, *p* < 0.001 for all comparisons) and in AP directions (*post hoc*, *p* < 0.0001 for all comparisons). ANOVA showed also a difference between trial repetitions for ML [*F*_(7, 147)_ = 16.8, *p* < 0.0001, η^2^*_*p*_* = 0.44] and AP directions [*F*_(7, 147)_ = 22.4, *p* < 0.0001, η^2^*_*p*_* = 0.52]. There was also a significant interaction between conditions and trial repetition for both ML [*F*_(21, 441)_ = 4.75, *p* < 0.0001, η^2^*_*p*_* = 0.18] and AP directions [*F*_(21, 441)_ = 7, *p* < 0.0001, η^2^*_*p*_* = 0.25]. Hence, the median frequency patently diminished with the successive trials, a sign of relative progressive prevalence of lower over higher body oscillation frequencies, particularly without vision. All the *post hoc* comparisons are reported in Supplementary Tables 3, 4. For both ML and AP directions, median frequency was different between the first and last trials under EC and EC-LT conditions (*p* < 0.001, for all comparisons). Under EO and EO-LT conditions, median frequency was not different between the first and the last trial (*p* > 0.09), except for AP EO-LT condition (*p* < 0.05).

The mean slopes of the regression lines fitted to the log-transformed values of the successive trials are reported in Figure 4C for the median frequency data of each sensory condition. ANOVA showed a difference in the slopes between sensory conditions [*F*_(3, 63)_ = 8.4, *p* < 0.0001, η^2^*_*p*_* = 0.29] and an interaction between ML and AP directions and sensory conditions [*F*_(3, 63)_ = 2.95, *p* < 0.05, η^2^*_*p*_* = 0.12]. There was no difference between ML and AP directions in the slope of the median frequency [*F*_(1, 21)_ = 1.2, *p* = 0.28]. For the EC and EC-LT conditions, the slopes were definitely decreasing (the white and black bars in **C**) in the successive trials, the slopes of the lines fitted to the EO and EO-LT data reached significance only for AP EO-LT. The slope of the median frequency in ML direction was different between EO-LT and the other conditions (*post hoc*, *p* < 0.05, for all comparisons). The slope of EC-LT was greater than that of the EO condition (*p* < 0.01) for both ML and AP directions. The slope was not different between EC and EO conditions in the ML direction (*p* = 0.4), but there was a difference between these two conditions in the AP direction (*p* < 0.01). Obviously, the adaptation trends in the median frequencies were different between no-vision and vision, while touch had a small effect on these trends. The difference between the amplitude of the profiles of the last (red) and first (black) trials reported in Figure 3, added to the increase in the amplitude of the low-frequencies, likely explain the relative decrease in the values of the median frequency of the last compared to the first trial.

### Effect of Trial Repetition on the Mean Level of the Spectrum of the Centre of Feet Pressure Excursions in the Different Sensory Conditions

The mean level of the spectrum for the subsequent trials in each sensory condition is reported for the ML and AP directions in the lower panels of Figures 4D,E. While the adaptation effect on the median frequencies was plain to see, there was no progressive decrease in the mean level of the spectrum in the ML direction and a moderate, albeit definite, progressive increase in the level of AP spectrum. The mean level of the spectrum was different between sensory conditions in both ML [*F*_(3, 63)_ = 65.7, *p* < 0.0001, η^2^*_*p*_* = 0.76] and AP directions [*F*_(3, 63)_ = 95.5, *p* < 0.0001, η^2^*_*p*_* = 0.82]. The mean spectrum level for the “stabilised” sensory conditions (EC-LT, EO, and EO-LT) was clearly smaller than with EC (*post hoc*, *p* < 0.0001 for all comparisons). ANOVA showed no significant effect of trial repetitions for the ML direction [*F*_(7, 147)_ = 0.38, *p* = 0.91], but an effect for the AP direction [*F*_(7, 147)_ = 4.9, *p* < 0.0001, η^2^*_*p*_* = 0.19]. There was no significant interaction between sensory conditions and trial repetition for either ML [*F*_(21, 441)_ = 1.4, *p* = 0.1] or AP directions [*F*_(21, 441)_ = 0.66, *p* = 0.87]. All the *post hoc* comparisons are reported in Supplementary Tables 5, 6. In general, there was no significant difference in the mean level of the spectrum along the ML direction between the first and last trials (*p* > 0.12), except for EC condition (*p* < 0.05). For the AP direction, there was a difference between the first and last trials (last > first) for all sensory conditions (*p* < 0.05, except EC-LT, *p* = 0.09). The slopes of the lines fitted to the log-transformed data of the mean levels of the spectrum are reported in Figure 4F. Slopes were generally close to zero in ML direction, but greater than zero in AP direction, indicating a progressive increase in the spectrum level in the sagittal plane. ANOVA showed a difference between ML and AP directions [*F*_(1, 21)_ = 20.3, *p* < 0.001, η^2^*_*p*_* = 0.49], a difference between sensory conditions [*F*_(3, 63)_ = 5.2, *p* < 0.01, η^2^*_*p*_* = 0.2] and an interaction between ML and AP directions and sensory conditions [*F*_(3, 63)_ = 4.5, *p* < 0.01, η^2^*_*p*_* = 0.18]. The *post hoc* analysis showed lower slopes in the ML than in AP direction (*p* < 0.01 for all comparisons) except for the EO condition (*p* = 0.14). For ML direction, the slope of the EC was different from that of EC-LT and EO conditions (*post hoc*, *p* < 0.05, for both comparisons), but not from EO-LT (*p* = 0.09). For the AP direction, the slope in the EO-LT condition was greater than that in the other sensory conditions (*p* < 0.01, for all comparisons). The slope in EC was also smaller than that in the EC-LT condition (*p* < 0.01). The differences between the amplitude of the profiles of the last (red) and first (black) trials reported in Figure 3 explain the relative constancy of the mean levels of the power spectra compared to the large decrease in the values of median frequencies because the adaptation-induced increase in the lower frequencies is compensated by the decrease in the higher frequencies.

### Distinctive Changes in the Mean Level of the Spectrum of the Frequency Windows

**FIGURE 5:**
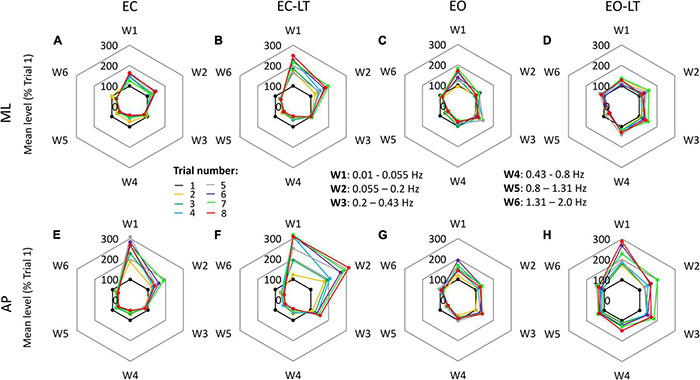
Changes in the mean level of the power spectrum in the six frequency windows, in which the full spectrum (from 0.01 to 2.0 Hz) was separated, for all trials and sensory conditions. Data are expressed as a percent of the spectrum mean level of the first trial (black dots at 100%) in each condition and direction (this prevents a quick comparison of the graphs between conditions). In general, for both ML **(A–D)** and AP **(E–H)** directions and for all sensory conditions, the mean level of the spectrum increased over the successive trials in the first two windows (W1 and W2), showed minor changes in W3, and decreased in the windows from W4 to W6.

There were similarities and differences in adaptation across conditions and directions of CoP excursion. The radar charts of Figure 5 show the progressive amplitude changes (successive trials are indicated in different colours) of the six frequency windows, for the four sensory conditions (from left to right) and for the ML and AP directions (top and bottom, respectively). Note that the levels of the spectrum are reported in these charts in *percent* of the level of the first trial (the inner black 100% hexagon) for each window, to render the changes in the higher frequency windows more conspicuous compared to their absolute values. This procedure allows to compare the different trials within a given sensory condition, but not between different sensory conditions or ML and AP directions. A major rise in the level of the spectra of the low-frequency windows occurred with EC, EC-LT, and EO-LT. Its percent increase compared to the first trial was prominent in W1 and W2 (from 0.01 Hz to 0.2 Hz) with EC-LT in the AP direction. Concurrently, definite reductions occurred in the high-frequency windows (W4–W6). These reductions were common to ML and AP directions. A further noticeable finding was the largely unaltered amplitude of the spectrum of W3, mostly common to all sensory conditions and directions.

**FIGURE 6:**
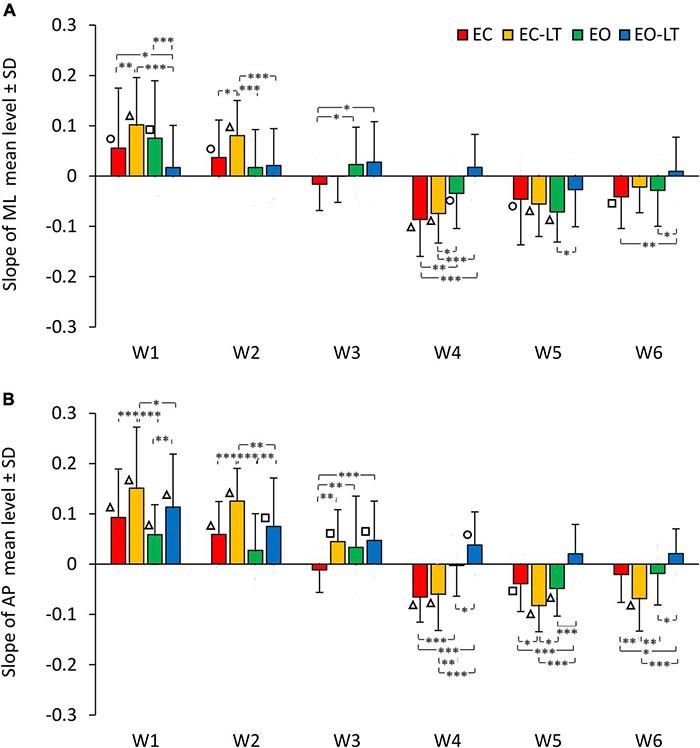
Adaptation rate in the frequency windows (Ws) of ML **(A)** and AP **(B)** spectra. The mean slopes of the lines calculated for each frequency window (W) are reported for ML and AP in the different sensory conditions (red, EC; yellow, EC-LT; green, EO; blue, EO-LT). For all sensory conditions, the slopes of W1 and W2 are always positive indicating an increment, during the trial repetition, in the mean level of the spectrum. In W3, the slope is not different from zero in the ML direction for all sensory conditions, while in the AP direction was positive, except for the EC condition, and different from zero only for touch (EC-LT and EO-LT). From W4 to W6, except for the EO-LT condition, the slopes are always negative indicating a reduction in the mean level of the spectrum during the trial repetition. Asterisks indicate significant differences between sensory conditions (*p < 0.05; ^**^p < 0.01; ^***^p < 0.001). Symbols indicate slopes significantly different from zero (◯*p* < 0.05; □*p* < 0.01; △*p* < 0.001).

The graphs in Figure 6 add information on the mean adaptation rates of the mean levels of the spectra of each frequency window in the four sensory conditions (same colour code as in Figures 2, 4) in ML (Figure 6A) and AP directions (Figure 6B). Sensory conditions [*F*_(3, 63)_ = 6.51, *p* < 0.001, η^2^*_*p*_* = 0.24], frequency windows [*F*_(5, 105)_ = 65.01, *p* < 0.0001, η^2^*_*p*_* = 0.76] and ML and AP directions [*F*_(1, 21)_ = 20.2, *p* < 0.001, η^2^*_*p*_* = 0.49] affected the adaptation pattern. Some patterns were common to all conditions, some were different. There was an interaction between ML and AP directions and frequency windows [*F*_(5, 105)_ = 2.44, *p* < 0.05, η^2^*_*p*_* = 0.1], between ML and AP directions and sensory conditions [*F*_(3, 63)_ = 3.26, *p* < 0.05, η^2^*_*p*_* = 0.13], between frequency windows and sensory conditions [*F*_(15, 315)_ = 8.17, *p* < 0.0001, η^2^*_*p*_* = 0.28] and between ML and AP directions, frequency windows and sensory conditions [*F*_(15, 315)_ = 2.5, *p* < 0.01, η^2^*_*p*_* = 0.11]. Specifically, some frequency windows were unaffected by adaptation, while some were deeply modified with growing or diminishing spectrum amplitude. The amplitude of the low-frequency windows (W1 and W2) had a definite increasing trend (positive slope) with trial repetition, W3 persisted almost unchanged with a slope not different from zero for all conditions in the frontal plane and minimally changed in the sagittal plane, the last three frequency windows progressively decreased (except with EO-LT).

Common patterns were the larger positive slope of the low-frequency windows in AP than in ML (*post hoc*, W1, *p* < 0.05 and W2, *p* < 0.01, except for EO-LT), and the generally negative slopes in W4–W6, similar between ML and AP (*p* > 0.07 for all comparisons except for EC-LT in W6, *p* < 0.01). Moreover, there was no statistical difference from zero in the slope of EO-LT condition in the frontal plane for all frequency windows, and a positive slope of the adaptation rate in the EO-LT in the sagittal plane for all windows up to W4. The results of the *post hoc* comparisons between sensory conditions in the distinct frequency windows are reported in Supplementary Table 7.

### Differences Between the Mediolateral and Anteroposterior Spectra of the Adapted Trials in the Different Sensory Conditions

**FIGURE 7:**
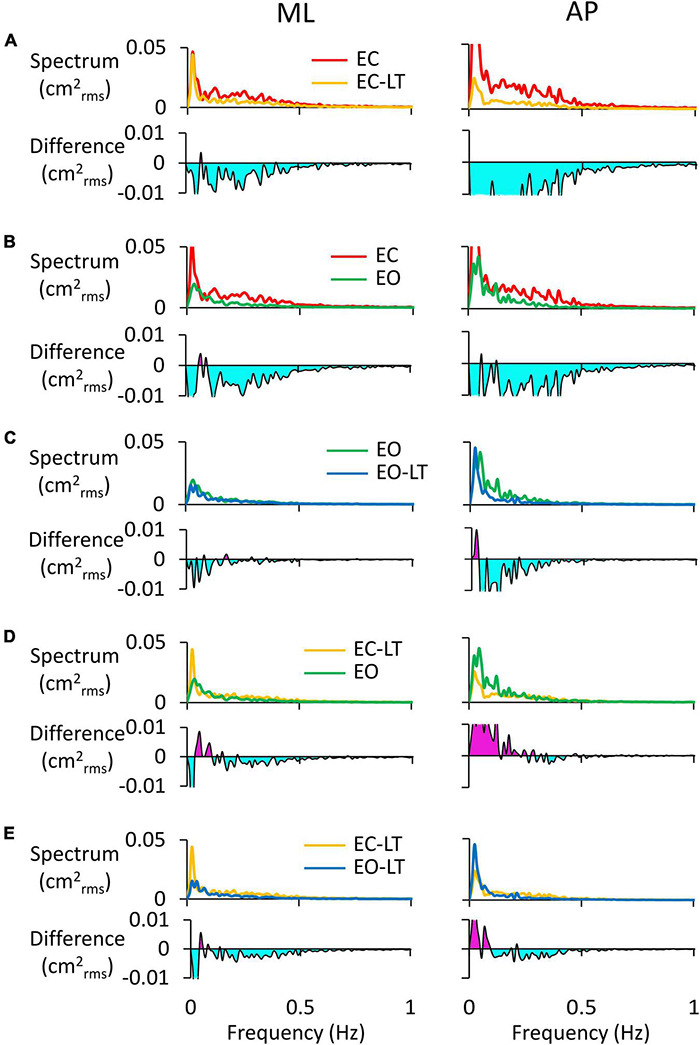
Comparisons of adapted trials between sensory conditions (coloured). In the upper traces of each panel, the mean power spectra of ML (left column) and AP directions (right column) of the last trial are reported and compared. In the lower traces, the difference between spectra is shown: **(A)**, EC-LT minus EC; **(B)**, EO minus EC; **(C)**, EO-LT minus EO; **(D)**, EO minus EC-LT; **(E)**, EO-LT minus EC-LT. Light blue areas highlight negative differences between the pairs of sensory conditions. The pink area in **(D,E)** indicates that the spectrum of the most stabilised condition (e.g., EO in **D** and EO-LT in **E**) is greater than the contrast condition (EC-LT in **D,E**).

The power spectra of the adapted (last) trials have been compared to each other between sensory conditions. For instance, the comparison of the spectra of the EC and EC-LT conditions would disclose the unique effect of touch in the absence of vision, while the comparison between EO and EO-LT would reveal the effect of touch in the presence of vision. In Figure 7, the left and right columns refer to the ML and AP directions, respectively. In each panel of Figures 7A–E, the upper traces show the superimposed profiles of the adapted spectra. The bottom traces show the amplitude of the differences between the spectrum profiles (the minuend and the subtrahend are indicated in the legend). In general, most adapted spectra showed substantial differences depending on the selected paired conditions, the ML or AP directions, and the range of oscillation frequencies.

In Figure 7A (EC-LT compared to no-touch EC), touch reduced both the ML (left) and the AP (right) adapted spectra. The differences were scattered across all frequencies (mostly below 0.5 Hz) but were much larger for AP than ML (AP > ML, compare the light blue areas in the lower traces of Figure 7A). Vision (Figure 7B, EC compared to EO) led to changes similar to those produced by touch in the EC adapted trial. Adding touch to vision (Figure 7C, EO-LT compared to EO), in the context of an overall much smaller spectrum level, slightly reduced the very low frequencies in ML direction in the adapted trial. Again, however, the reduction in the amplitude of the power spectrum was of some importance for the AP excursions, where differences were recurrent also at higher frequencies. In other words, the reduction in the mean level of the spectrum by adaptation was more substantial in AP than ML when touch was present. The comparison of the adapted trials with touch no-vision and with vision no-touch (EC-LT vs. EO, Figure 7D) yielded modest differences in the ML direction. The touch no-vision adapted trials showed a lower amplitude of the spectrum up to 0.2 Hz in AP direction compared to EO. Figure 7E shows the difference between the adapted trials with and without vision in presence of touch (EC-LT vs. EO-LT). With touch and vision (EO-LT) compared to touch no-vision (EC-LT), adaptation reduced the spectrum between 0.1 and 0.5 Hz in both ML and AP direction. At the lowest frequencies, the spectrum was larger in AP than ML directions.

### Force of Finger Touch Across Trials

**FIGURE 8:**
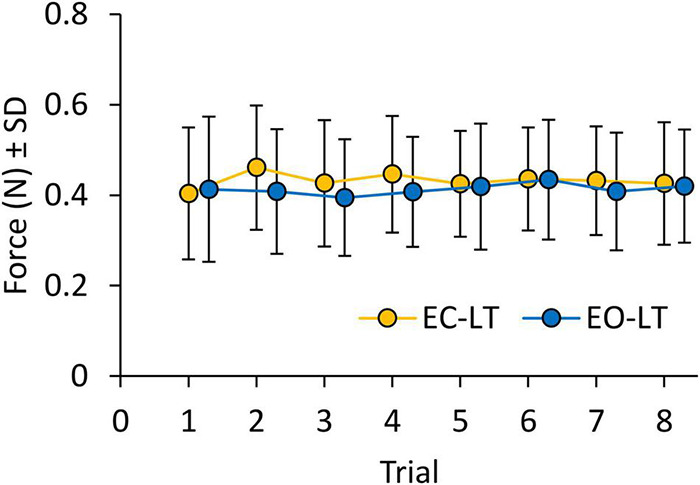
Force applied on the touchpad during trials (means of all subjects). Force was not different between EC-LT (yellow symbols) and EO-LT (blue symbols). In both conditions, the force never exceeded1 N and remained constant across trials.

Figure 8 shows the mean level of the force applied by the subjects on the touchpad during the EC-LT and EO-LT trials. The mean force never exceeded 1 N, despite non-negligible variability across subjects. ANOVA showed no difference between conditions [EC-LT vs. EO-LT, *F*_(1, 20)_ = 1.1, *p* = 0.31] and no main effect of trial repetition [*F*_(7, 140)_ = 1, *p* = 0.46]. There was also no significant interaction between conditions and trial repetition [*F*_(7, 140)_ = 1.3, *p* = 0.26]. Therefore, the observed changes in the geometric and spectral measures were not related to a graded change in the fingertip force exerted during the subsequent trials.

## Discussion

### Body Sway Adaptation to the Repeated Standing Trials

We had postulated that a process of postural adaptation would take place, also in the absence of reactions to external artificial stimulation(s), and reflect a progressive involvement of higher centres (Mierau et al., 2015; Kaulmann et al., 2020). We leveraged the use of the modulations in the frequency of the power spectrum of the CoP excursions (Schumann et al., 1995), following the approach exploited in several studies on balance adaptation to postural disturbance (Loughlin et al., 1996; Kiers et al., 2015; Borel and Ribot-Ciscar, 2016; Assländer et al., 2020). In accordance with our assumptions, we have seen that adaptation occurs during repeated, prolonged standing-on-foam trials. This adaptation affects both geometric variables (path length and sway area) and spectral markers (median frequency and mean level of frequency spectrum) of the CoP excursion. Where significant differences were observed, the adaptation impact had an effect size greater than the minimum effect size detectable based on our sample of 22 participants.

We have found peculiar features of adaptation (meaning here both a decrease *and* an increase in the value of distinct variables) in the oscillations of the CoP, with features depending on availability of vision and touch. The changes in the responsiveness to the closely recurrent exposures to stance trials were contingent on the metric considered and on the direction (ML or AP) of the CoP excursion. The power spectrum of the oscillations showed distinct features in the different sensory conditions, and the mean level of distinct frequency windows of the spectrum changed over the trials depending on the conditions. Finally, the adapted behaviour at the end of the trial sequence did not overlap for all sensory conditions, indicating that there is no unique, common “default” or “optimal” adapted behaviour, as a necessary outcome of trial repetition.

Several new findings of the present study deserve attention. (1) Adaptation is present under some but not all sensory conditions. (2) Adaptation does not eventually lead to a stable state, as when a ball in a parabolic bowl of any shape (here, the sensory conditions) is released from some initial height and allowed to roll back and forth inside the bowl. (3) Adaptation rate is not necessarily related to the amplitude of the given variable in the first trial. (4) Adaptation reduces the amplitude of some sway variables and increases that of others. (5) CoP path length generally decreases while sway area generally increases. (6) In the spectral dimension, the median frequency generally decreases, whereas the mean level of the spectrum tends to increase over time, mainly in the AP direction. (7) Importantly, adaptation differently affects distinct frequency ranges, whereby the low frequencies increase in amplitude, middle-range frequencies remain constant, high frequencies decrease. (8) The adaptation process differently affects the mean level of the spectrum and its adaptation rate in the frontal and the sagittal planes.

The subjects’ report about absence of fatigue or “muscle tension” at the end of the session, and the declared negligible physical effort for standing, rule out the possibility that fatigue plays a role in the adaptation by increasing the CoP oscillations (Nardone et al., 1997; Bisson et al., 2012; Bermejo et al., 2015). Incidentally, an increase in the high-frequency band was observed with leg muscle fatigue (Bizid et al., 2009), but is not seen in the present study. Moreover, the adaptation process was not accompanied by matching changes in haptic input over time, since the force applied to the touchpad was constant across the repeated trials, regardless of vision availability. In a sense, the absence of the stabilising visual input was not compensated by a stronger haptic inflow, in keeping with the conclusions by Garbus et al. (2019).

### The Adaptation Process Occurs in the Absence of Repeated Balance Perturbations

It might appear extravagant to address the adaptation process of standing upright without administering external or artificial perturbations. Rarely if ever it happens that people stand in place for minutes, except perhaps when standing at attention if you are a soldier (normally on a non-compliant base of support). Anyhow, for that matter, repeated perturbations hardly ever come about in real life. However, both approaches to this issue are valuable, because they deal with the general theme of changes in motor behaviour taking place with task repetition. In accordance with our expectations, an adaptation process occurs during repeated, prolonged standing-on-foam trials. In particular, standing on foam induces self-imposed (or internally generated) perturbations of the stance that need to be counteracted by reactions to the elicited stimuli. Both foam and perturbations imply balance threat, reflex responses, descending modulation, and both ultimately call for mastering balance (Tjernström et al., 2002, 2005; Clair et al., 2009). But standing on foam does not imply any degree of preparatory or expectancy activity that may interfere with responses to perturbations.

Just to mention distant examples of “adapting” behaviours, impressive long-term plasticity helps control balance when the underpinning neural processes slowly deteriorate over time (see Tighilet and Chabbert, 2019; Barmack and Pettorossi, 2021). On a very short time scale, post-effects of a particular sensory condition are observed on shifting to a different condition (Hay et al., 1996) or are produced by sensory reweighting after attenuation of one modality (Billot et al., 2013; Honeine and Schieppati, 2014), or simply modify postural adjustments after a single training session of a catching task (Kanekar and Aruin, 2015). What triggers adaptation, whether it is dependent on predictions of balance threats or on the capacity of reweighting the sensory information, or else on explicit learning is a matter of controversy (Peterka and Loughlin, 2004; Assländer et al., 2020; Bakker et al., 2021; see Rothwell et al., 2021, for an excellent short account of neural adaptation recourses). The present investigation claims to be a preliminary methodological approach to such a complex question.

A decrease in body sway occurs with trial repetition when standing on a solid base of support in the absence of vision, but not when vision is available (Tarantola et al., 1997; Reed et al., 2020). Drawing on that former finding, we have investigated here the balance adaptation process of young healthy subjects during standing on foam, having in mind that presence or absence of touch and vision can entail different “attractors” (Lee et al., 2018) and disclose uncharted modulations of central integration processes (Sozzi et al., 2021). The foam was selected because it is more demanding compared to standing on a solid base of support (Allum et al., 2002). Foam challenges control because the reaction of the compliant surface to muscle action is unfamiliar and unpractised and enhances the level of attention and voluntary corrections (Di Fabio et al., 1990).

### Eyes Closed, Without, and With Touch

At variance with quiet stance on a solid surface, where geometric markers of body oscillation are almost superimposable without (EC) and with vision (EO) (Sozzi et al., 2021), sway on foam is much larger EC than EO. Adaptation is clearly observable with EC, where the body oscillations, measured by CoP path length, are the largest and progressively diminish (Jeka and Lackner, 1995), but remain larger at the end of the adaptation period, compared to the other sensory conditions. This suggests that the absence of visual information can be compensated—not completely—by the progressive up-weighting of the remaining senses (proprioception, vestibular, plantar cutaneous) (Bernard-Demanze et al., 2004; Šarabon et al., 2013a; Goodworth et al., 2014). As observed under different conditions, i.e., perturbation of stance by a mobile platform (Sozzi et al., 2016), the adaptation is obvious with EC (again not with EO) and is not immediate.

With EC, adaptation entrains a pronounced reduction of the median frequency of the spectrum (for both ML and AP directions), while the mean level of the spectrum remains constant in the ML direction and slightly increases in the AP direction. In other words, the excursions of the CoP become slower, but the size of the surface covered by the wandering of the CoP remains large across the repetitions. It seems that accuracy in maintaining the CoP within a limited space of the support base is not required when standing on foam. It might be useless to strictly control the CoP excursion when no steps or gait initiation are planned, or in the absence of an explicit instruction to stand as still as possible (Bonnet, 2016), or else because muscle co-contraction in addition to being costly (Houdijk et al., 2015) would be unsafe. Rather, exploiting the redundancy of the body segments’ degrees of freedom can enhance the chance of finding a safe posture (Rasouli et al., 2017) or several safe postures over time despite the absence of vision.

These features are broadly common to EC-LT when touch is added in the absence of vision. Sway is reduced with touch (EC-LT) to values a little larger than with vision. However, the rate of decrease in path length is analogous to that in EC condition, even though path length is initially only two-thirds of that with EC. This relative difference remains in the final trials as well. Much as for EC, the sway area with EC-LT slightly increases in size and the median frequency decreases with adaptation. Hence, touch eliminates a certain constant amount of oscillation amplitudes and (high) frequencies, while the adaptation rate itself is set by the absence of vision. The mechanisms through which touch reduces sway are not flagrantly time-dependent. Kaulmann et al. (2020) also observed that light touch did not reduce the time constant of postural compensation following a perturbation. They suggested that owing to the overall smaller sway with than without touch, an invariant time constant would nonetheless allow reaching a steady-state earlier with than without touch.

### Eyes Open, Without, and With Touch

With vision (EO), the progressive decrease in path length is absent. In the beginning, the path length is much smaller than with EC (<50%) and remains constant. With touch added to vision (EO-LT), the path length is the shortest and remains constant over time as well. Sway area is also small, but slightly and steadily increases. The mild rise is accompanied by an increase in the mean level of the spectrum in the very-low-frequency range (<0.1 Hz) and by a minor reduction in the value of the median frequency. It has been shown already that vision compared to no-vision reduces the sway frequencies below 1 Hz (Dichgans et al., 1976; Mezzarane and Kohn, 2007; Sozzi et al., 2021). However, the lowest frequencies (W1 and W2) do not further decline with the adaptation, rather they relatively build up over the successive trials instead (more in AP than ML). Perhaps, with touch and vision (EO-LT), subjects feel safe and free to sway thanks to the haptic input, meaning that they can change freely the activation turnover across many relevant postural muscles. While this seems to indicate evidence of a reweighting of the haptic input itself, the concurrent slow increase in the sway area (EO-LT) might indicate a shift to progressive better exploitation of the exploratory behaviour (Carpenter et al., 2010; Fabre et al., 2020). This could also contribute to preventing receptor and muscle fatigue.

### The Contribution of Vision and Touch to the Adaptation

Initial values of body sway with vision or touch are comparable to those found previously (Rogers et al., 2001; Wing et al., 2011; Sozzi et al., 2021). We confirm here that both vision and touch exert a powerful stabilising effect even when standing on foam and that adaptation is present under some but not all sensory conditions. The adaptation is prominent without vision in the geometrical markers and in the median frequency. Despite the differences in the amplitude of the spectra and in the median frequency, the adaptation rates are similar in the EC and EC-LT. Adaptation rates are also similar between EO and EO-LT, but their value is much slower without vision, possibly because of a floor effect given by the low mean level of CoP excursion and low median frequencies of the spectra. During the adaptation period, the spectrum shifts toward the low frequencies (W1 and W2, in ML and AP directions). Touch, much as vision, progressively reduces the amplitude of the spectrum in the high-frequency range (W4–W6) in both ML and AP directions (Riley et al., 1997; Rogers et al., 2001). Whereas, touch speeds up the rise of the low frequencies compared to the same visual condition without touch. Touch reduces by a constant amount path length, sway area and mean level of the spectrum compared to no-touch, regardless of visual condition (EC-LT and EO-LT). This “offset” is large with EC and minimal with EO, but in both cases, it does not vary with adaptation. It would be safe to conclude that the process of postural adaptation is set when vision is not available and that, with vision, adaptation is almost ineffectual. Oddly enough, with touch and vision, all geometrical and spectral markers are manifestly diminished compared to EC, but during the adaptation process, a slight but significant increment in these markers is observed as if the stabilising effect of vision and touch would be down-weighted. The rate of this increment is slightly greater with than without touch. Perhaps, in the adaptation period, a trade-off is reached whereby the postural control system accepts a minor stabilisation (in the sagittal but not in the frontal plane) in exchange for a more relaxed attitude and less attention spent in the control of the finger force. Likely, touch acted mainly as a task demand, i.e., vision set by trial and error the stabilisation process while touch was a “supra-postural task” exploiting but not contributing to the stabilisation process (Lee et al., 2018).

### Non-identical Adaptation Along the Frontal and Sagittal Planes

The control of balance in the frontal and sagittal plane is peculiar (Day et al., 1993; Winter et al., 1996) and conditional on the interaction of two independent postural sub-systems, the synergic action of which complies with the demands of a precision task (here represented by the maintenance of the equilibrium, with or without the light touch) (see Balasubramaniam et al., 2000). The excursions of the CoP in the ML and AP directions are produced by coordinated activation of different muscles (about the hip and the ankles, respectively) (Winter et al., 1996; Zhang et al., 2007). Singh et al. (2012) showed that visual information focuses the control of sway in AP direction at almost all frequencies, whereas a foam surface rather affects sway in ML direction at the middle (from about 0.6 to 0.7 Hz) and high (from about 2 to 7 Hz) frequencies. Importantly, sway amplitude in the frontal plane is proportional to the threshold of vestibular encoding of lateral body translation (Karmali et al., 2021), and ageing affects both the vestibular and the balance systems (see Wagner et al., 2021). When standing on foam, the head is stabilised better in the frontal than the sagittal plane in healthy subjects, showing accurate control of hip motion in roll (Fino et al., 2020). Hence, any effect of the adaptation on the ML or AP excursions seems to be of interest in the light of potential interventions.

Adaptation manifests itself along both the AP and ML directions in all sensory conditions. This is shown by a decrease in the median frequency (EC and EC-LT) and by the increase in the mean spectrum level, especially in AP. This increase is caused by the shift of the oscillation frequencies toward very low values (<0.2 Hz, W1 and W2) and a decrease in the medium-high frequencies (0.3–2 Hz, corresponding to about W4–W6). The increase in the frequencies lower than 0.1 Hz confirms the conclusions of Rasouli et al. (2017) and Yamagata et al. (2019) about the presence of slow “drifts.” We do not know why the slow drifts build up with adaptation to a greater degree in the sagittal plane, but this is probably related to the foot-ankle architecture, whereby larger and slower torques are allowed in the sagittal than the frontal plane (Murray and Sepic, 1968; Pai and Patton, 1997). This favours a “safer” fore-aft balancing strategy by the slow ankle or hip rotations in the sagittal plane (Ogaya et al., 2016), likely assisted by the elastic properties of the foam. With touch, the spectrum amplitude remarkably diminishes, but part of the “drift” effect in the AP direction that builds up with adaptation can be connected to changes in the tone of the axial muscles (Johannsen et al., 2007; Franzén et al., 2011; Wing et al., 2011). This would be favoured by the position of the haptic device just in front of the subjects (Rabin et al., 1999). With vision, the adaptation rate of the spectrum amplitude is smaller in ML than in AP direction, likely because the large distance between the feet minimises the role of vision in ML balancing (Day et al., 1993; Šarabon et al., 2013a) and assures a safe ML sway from the onset of the trials.

### Potential Processes Underpinning Adaptation

The adaptation process of body sway, described here by geometrical and spectral analysis, can hardly be traced to a few definite sources. As mentioned, foot plantar and proprioceptive input from many muscles must be enhanced by standing on foam. In a sense, standing on a compliant base of support might have analogies with some sensory augmentation conditions, and adaptation to this state implies several different operations (Sienko et al., 2018). At least some of the mechanisms implied in the adaptation to repeated predictable perturbations should not be disregarded. Stretch reflex modulation is a candidate, and changes in its excitability have been shown during adaptation processes (see Rothwell et al., 1986; Schmid and Sozzi, 2016; Sozzi et al., 2016; Mrachacz-Kersting et al., 2019). Here, the rate of the adaptation with eyes closed reminds that observed in young subjects exposed to continuous predictable perturbation of stance by sinusoidal AP displacement of the supporting platform (Sozzi et al., 2016). In that case, as much as in the present investigation, a progressive decrease in the leg muscle activity was present with eyes closed but not with eyes open, where muscle activity was smaller.

In the absence of vision, the nervous system would exploit a sensory inflow to which it is unaccustomed (standing on foam), by initially increasing muscle stiffness (Winter et al., 1998; Craig et al., 2016) to later learn to appropriately reweigh and select the helpful and cancel the disturbing information. We suggest that when subjects realise the absence of a real risk of toppling over by standing on foam [as well as during continuous predictable perturbations as in Castro et al. (2019) in older adults] they feel safe, reduce postural muscle co-contraction, and energy expenditure (Welch and Ting, 2014) and accept larger, slower CoP excursions (Karmali et al., 2021). This would lead to a reduction of the median frequency of the spectrum. Moreover, the progressive increase in the amplitude of the low-frequency excursion is compatible with the high vestibular thresholds at these frequencies (Valko et al., 2012), as if the adaptation process shifted body oscillations toward those at which the vestibular input can be best exploited (Karmali et al., 2014). Of note, in spite of a substantial difference between the studies, activation of the midline fronto-central cortex is associated with adaptive behaviour to repeated postural perturbations unpredictable in timing much as occurs when standing on foam (Mierau et al., 2015; Varghese et al., 2019).

In our experiment, adding touch to EC further enhances the shift toward the low frequencies of the spectrum. Light-touch would “optimise” the processing of the relevant sensory information, both from the body and the touching limb as well, leading to sparing of motor actions around 0.4 and 2 Hz, possibly by enhancing coordination of muscle actions (Albertsen et al., 2012). Consequently, the control of stance moves from a strategy whereby a high oscillation rate dominates postural control to one where slower CoP sway predominates (Rasku et al., 2012).

We would speculate that a sway “threshold” exists, below which the adaptation mechanisms would hardly (EO, with or without LT) be called into action. For example, in our case (see Figures 2, 4), the threshold should be just below 0.2 Hz for median frequency values and just below 2 m (over 90 s) for path length. Which is or are the responsible excessive-sway-detecting receptors or the brain centres possessing some kind of “velocity-storage” mechanisms would require a different experimental and analytical approach (Jeka et al., 2004; Assländer and Peterka, 2016; Appiah-Kubi and Wright, 2019).

### Rambling and Trembling

The reduction of path length is accompanied by an increase in the amplitude of the low-frequency displacements of the CoP with a reduction in the median frequency of the full spectrum. A description of body sway has identified two processes under the names of rambling and trembling (Zatsiorsky and Duarte, 1999, 2000). Rambling has been suggested to be the expression of supraspinal control, while trembling attests spinal control. During prolonged standing, large-amplitude changes in rambling may be observed. Adaptation on foam could be instructed by the same processes originally described for stance on solid ground (Duarte and Sternad, 2008). The “trembling” component might imply inordinate CoP excursions, not immediately filtered by the supra-spinal modulating influences. The high frequencies might be more unfavourable than serviceable for the “exploration” task. The progressive reduction in trembling can avoid the blurring cutaneous and proprioceptive inputs from the feet and legs, and reduce the neural and muscle cost. However, the median frequency diminishes to only about half of the initial values without vision, where adaptation is obvious and declines more smoothly with vision (except with vision *and* touch in the ML direction, where no adaptation is obvious). Therefore, a certain “share” of trembling is present and continues to control sway and help equilibrium maintenance (Yamamoto et al., 2015; Gerber et al., 2022), probably because difficult standing tasks favour automaticity (Haith and Krakauer, 2018) compared to tasks requiring less cognitive involvement (Hsiao et al., 2020; Leung et al., 2021). The shift from trembling to rambling would be controlled and checked by the cerebellum and higher centres (Reynolds, 2010; Colnaghi et al., 2017; Patikas et al., 2019). Anyhow, we cannot leave out the intriguing observation that a certain frequency range (∼W3) was largely unaffected by the adaptation. This frequency is intermediate between rambling and trembling, where the former vanishes and the latter starts to grow (Zatsiorsky and Duarte, 1999). Further, as the lower frequencies increased in amplitude, higher frequencies decreased with adaptation. The unvarying W3 window shows that adaptation is not a generalised depression of some collective activity, but reflects a hard-wired neural mechanism. There is an edge in the low-frequency CoP oscillations, whereby slow “rambling” displacements overcome fast, small, higher-frequency “trembling” components (Zatsiorsky and Duarte, 2000; Mochizuki et al., 2006; Yamamoto et al., 2015). Certainly, rambling becomes progressively more important during the adaptation process described here. This occurs also with EO and EO-LT, where adaptation is less obvious, and where the high frequencies are less represented than without vision (EC and EC-LT). Interestingly, older subjects show reduced “trembling” and increased “rambling” frequencies (Šarabon et al., 2013b), much as happens with younger subjects during the adaptation process.

### The Shift to Low-Frequency Oscillations Does Not Reflect Enhanced Automaticity

Our subjects made deliberate efforts to control balance, certainly at the beginning and throughout the standing trials as well. During the adaptation period, they would continuously seek to anticipate a forthcoming instability and produce preparatory or expectancy activity, in addition to controlling instability. However, no subject could trace any distinct voluntary activation of selected postural muscles when asked at the end of the session. In a sense, they seemed to implicitly learn to cope with the compliant base of support before the end of the trials.

The stabilising effect of touch is in keeping with its capacity to modulate the responses to unanticipated perturbation of stance (Martinelli et al., 2015), an effect accompanied by a decrease in muscle stiffness and prevalence of reciprocal activity in antagonist’s muscles (Sozzi et al., 2013; Dos Santos et al., 2019; Kaulmann et al., 2020). Light-touch with the constraint of keeping force level below 1 N is a precision task, and a dual-task might be elicited (Rabin et al., 1999; Chen and Tsai, 2015). Under different experimental conditions, sensory attention tasks modify the integration of proprioceptive input into the motor cortex, modulating the cortical learning processes (Bolton et al., 2011; Rosenkranz and Rothwell, 2012; see for a recent review, Dijkstra et al., 2020). In our case, light-touch required some attention, even if quite different from an explicit arithmetic task (Vuillerme et al., 2006; Honeine et al., 2017; Lee et al., 2019). A decreasing contribution from the visual system, with a concurrent increase in contribution from the cerebellum and vestibular system in dual-task conditions, represents a shift from controlled to automatic postural behaviour (Lang and Bastian, 2002; St-Amant et al., 2020). The automatic behaviour would show a greater contribution of higher frequency bands in cognitive task conditions, though (Potvin-Desrochers et al., 2017; Richer and Lajoie, 2020), which is different from what we found with adaptation.

The interference of a dual-task with balance control is an open issue (Chen and Tsai, 2015; Bayot et al., 2018; Costa et al., 2021), including the simultaneous performance of a cognitive task with finger-touch stabilisation (Lee et al., 2018; Dos Santos et al., 2019). Here, touch does not increase sway compared to no-touch (without and with vision). Hence, no dual-task property should be conferred to the standing behaviour by light touch, at least not of greater moment than free viewing, which likely requires some attention as well (Bonnet et al., 2017). Possibly, the control of stance is prioritised when standing on foam (contrary to the “posture-second strategy,” see Bloem et al., 2006 in a different context), or else maintaining the light fingertip force is an easy task, as shown by the lack of changes over time in the fingertip force produced. In addition, a light touch is strongly stabilising in itself, thereby likely requiring a minor level of attention devoted to the control of standing upright.

### The Function of the Adaptation

There is no doubt that task repetition leads to improved stance control, for example, assessed behaviourally, where the “time in balance” increases with short-term training (Schedler et al., 2021). Getting closer to the underpinning mechanisms, we suggest that, when subjects stand on foam, the brain soon realises that minimisation of the body displacement *per se* is not an efficient way of coping with the critical condition (Kiemel et al., 2011). Conversely, the goal would be to set an oscillation amplitude compatible with minimal but *effective* muscle activation. In the end, a passive rigid body, even with a non-point-like base of support, falls more easily on a compliant than the solid base of support, whereas a continuous excursion of the CoP allows appropriate activation of selected muscles (be it reflex or voluntary) to create the necessary torques for adaptively controlling the excursions of the centre of mass. The reasoning is in keeping with several previous findings, based on theoretical and experimental approaches, which point to the inadequacy of stiffness *per se* to maintain equilibrium (Morasso and Schieppati, 1999; Moorhouse and Granata, 2007; Kiemel et al., 2011; Gorjan et al., 2019; Nandi et al., 2019). As a consequence, the oscillation frequencies gradually move toward low values *and* the high frequencies tend to disappear, while the overall level of the spectrum does not decrease but rather increases because of the large contribution of the low frequencies. This pattern is roughly common to the four sensory conditions, naturally graded to the overall amplitude of the spectra (e.g., quantitatively smaller with EC-LT than EC). Notably, even where the markers of adaptation are modest (as path length or median frequency in the stabilised EO and EO-LT conditions), a certain increase in the low frequencies is still present in the adapted trials, while the higher frequencies change little or diminish. It seems safe to conclude that adaptation modifies the quality of the oscillation, which becomes greater in amplitude but slower, so that balance control shifts from a stiff attitude to a more relaxed attitude and slower motion (Karmali et al., 2021). These conclusions do not seem to contradict a hypothesis put forward by Cherif et al. (2020). Their experimental setup is quite complex, but it might be more close to the unsophisticated foam-standing protocol, inasmuch as it delivers perturbations of the base of support to which subjects must react producing a focused corrective muscle activity. Their findings show that learning a force-accuracy control mode, producing minimisation of acceleration, was more effective than minimising sway by stiffness control.

### Differences in the Adaptation Pattern in the Geometrical and Spectral Measures

In conformity with published data (Tarantola et al., 1997), where subjects stood for repeated trials without vision on a solid base of support, reduction of path length over time occurs here when standing on foam with EC. About the same decrease is observed when touch is added to EC, despite an overall shorter path length. Sway area shows a trend over time as well, whereby its value moderately increased in the stabilised conditions (EC-LT, EO, EO-LT). This increase in sway area is not obvious with EC, where the area is by far the largest of all conditions. Therefore, assuming and not granting that both path length and sway area are considered appropriate tools for judging postural stability (Danna-Dos-Santos et al., 2008), these do not appear to be the most apposite markers for addressing the various aspects of adaptation of stance over time.

Moving to the spectral analysis, a different picture emerges. A clear-cut reduction in the median frequency of the full spectrum occurs with trial repetitions for the CoP oscillations along with both the frontal and the sagittal plane. The median frequency drops from more than 0.3 Hz to less than 0.2 Hz with EC and EC-LT, without notable changes in the mean level of the spectrum in the ML direction. The changes in the median frequency originate in an increased amplitude of the low- and a decrease in amplitude of the high-frequency windows, respectively. The findings suggest that conventional parameters, such as sway length (or velocity) and amplitude (Hufschmidt et al., 1980), do not provide sufficient information regarding a person’s ability to maintain an upright stance (see Gerber et al., 2022).

### Limitations

We have restricted the analysis to the spectral frequencies below 2 Hz, as we did in a recent paper (Sozzi et al., 2021). The spectrum level in the 0.01–2 Hz range corresponds to about 98% of the entire spectrum from 0 to 70 Hz. Our choice was in line with other studies that have limited the analysis to this range (Hayes, 1982; Day et al., 1993; Zatsiorsky and Duarte, 1999; Rougier and Farenc, 2000; Mezzarane and Kohn, 2007; Zhang et al., 2007; Demura et al., 2008; Mahboobin et al., 2009; Halická et al., 2014; Kanekar et al., 2014). Others have extended the range of interest up to higher frequencies. Some authors have posited that frequencies in a certain range (around 1.5–6.5 Hz) reflect the somatosensory contribution to balance control and represent the “moderate” band (Taguchi, 1977; Krafczyk et al., 1999). The lower band would express the contributions from the cerebellum (Diener et al., 1984), the very-low bands from the vestibular and the visual systems (Soames and Atha, 1982; Chagdes et al., 2009). We admit that the information on the exact frequencies at which distinct effects of diverse sensory input occur has not been acquired here, even if tackled by Sozzi et al. (2021) for vision and touch. The absence of recording of electrical activity from the many muscles potentially contributing to the CoP oscillations prevents a direct match of the changes in the spectral frequencies to the modulation of muscle activity.

The adaptation process has been studied by repeating 90 s standing trials with a short interval between trials not superior to half a minute, intended to soothe the subjects and discontinue peripheral processes like receptor adaptation or muscular fatigue. The 90 s trial duration and the number of successive trials has little correspondence with previous studies, possibly leading to incongruences in the findings (Cofré Lizama et al., 2016). However, the relatively long duration of the acquisition for each trial seemed appropriate based on previous research (Winter et al., 1998; Michalak and Jakowski, 2002; Sozzi et al., 2021). This epoch was not further divided into time segments and formally analysed to see if an adaptation occurs between the first and last part of the same trial. A related unanswered issue is the duration and amplitude of the adapted effects and the capacity to exploit learning for coping with difficult tasks (Schedler et al., 2021). In other words, we do not know whether and how post-effects of adaptation in sway or spectral measures fade over time.

In addition, there was some inter-subject variability in all sway metrics. Whether this reflects idiosyncratic postural sway in different subjects (Di Berardino et al., 2009; Yamamoto et al., 2015; Sakanaka et al., 2021) is not easy to tell based on our findings. In addition, we have not clustered the subjects into groups presumably showing different visuo-postural dependency (Lacour et al., 1997; Rasku et al., 2012) or differences in the body resonant frequency (Tarabini et al., 2014). It is also possible that different subjects became more or less tired out toward the end of the trials, despite none complaining of fatigue or dizziness, or requested to stop the trial, or asked for a longer rest period to be granted between trials. It must also be mentioned that only young adults but not younger or older subjects were investigated in this study. By exploiting a frequency-based analysis, Pauelsen et al. (2020) have recently shown issues in postural control in older adults with fall-related concerns and declining strength.

## Conclusion and Perspectives

The present investigation exploited the use of the frequency analysis of the CoP time series in a protocol implying prolonged standing on foam. The emerging picture is that repetition of stance trials leads to definite modulation of the standing behaviour. This consists of distinct but moderate changes in path length or sway area, and in qualitative and quantitative changes in the frequency content of the sway and in the amplitude of the frequency spectra.

The adaptation pattern reflects the current sensory conditions. Sway area increases over time, particularly in the stabilised conditions (EC-LT, EO, EO-LT) whereas the median frequencies of oscillation move toward low values, particularly without vision (EC and EC-LT). This occurs by enhancing the relative amplitude of the very low frequencies and reducing the higher frequencies over time. Throughout this process, the control of balance would be shifted from the lower to the higher nervous centres, with the aim to functionally incorporate the integrative capacities of the cortex and resolve the sensory ambiguity (Lhomond et al., 2021). In the sagittal plane, the mean level of the spectrum slowly increases in amplitude, regardless of its initial value, more than in the frontal plane, concurrently with a steeper rate of increase of the low-frequency windows. The different effects on the frontal or sagittal plane suggest that standing subjects can implicitly learn how to recruit the optimal strategies for controlling unstable stances. Of note, balance in the frontal plane is precarious in elderly subjects (Lord et al., 1999), where multiple sensory conditions can degrade the postural control (Morrison et al., 2016; see Paillard, 2021). These data give a rationale, though still preliminary, for explaining the results of training balance on a compliant support base (Strang et al., 2011) compared to other training modes (see Taube et al., 2008; Hirase et al., 2015; Nagy et al., 2018; König et al., 2019). If our paradigm captures some of the underlying causal mechanisms of adaptation, then adaptation features can become a standard marker of deficits in balance control in various populations at risk of falling (Schinkel-Ivy et al., 2016; Ruffieux et al., 2017). Understanding the interaction of balance control with the sensory condition and time has clinical implications. For instance, it would help investigate whether the adaptation rate differs where the postural disorders originate from peripheral neuropathy or a central condition (Asan et al., 2022). In this light, it is notable that patients with Parkinson’s disease show undamaged central processing of haptic cues and vision despite their motor problems (Rabin et al., 2013; Engel et al., 2021), even if their adaptability to prolonged standing seems to be impaired (Moretto et al., 2021). Further, challenging balance under the manifold and combined sensory states (Taube et al., 2007; Allison et al., 2018) rather than aiming at enhancing muscle strength (Thompson et al., 2020; Ramachandran et al., 2021) might exert positive effects in persons with precarious balance and older people.

## Data Availability Statement

The original data supporting the conclusions of this study are included in the article. Further inquiries can be directed to the corresponding author upon reasonable request.

## Ethics Statement

These studies involving human participants were reviewed and approved by the Ethics Committee of the IRCCS Istituti Clinici Scientifici Maugeri SB. The participants provided their written informed consent to participate in the present study.

## Author Contributions

MS conceived the idea for the study. SS performed the recruitment of participants and the collection of data. SS and MS performed the data analysis and drafted the article. Both authors approved the submitted version.

## Conflict of Interest

The authors declare that the research was conducted in the absence of any commercial or financial relationships that could be construed as a potential conflict of interest.

## Publisher’s Note

All claims expressed in this article are solely those of the authors and do not necessarily represent those of their affiliated organizations, or those of the publisher, the editors and the reviewers. Any product that may be evaluated in this article, or claim that may be made by its manufacturer, is not guaranteed or endorsed by the publisher.
