# Supplementary material for: Balance Adaptation While Standing on a Compliant Base Depends on the Current Sensory Condition in Healthy Young Adults
Source: Front Hum Neurosci. 2022 Mar 25;16:839799. doi: 10.3389/fnhum.2022.839799 (PMC8989851; doi:10.3389/fnhum.2022.839799)
Supplement: Supplementary file 1 [file Table_1.DOCX]

***Table 1.*** *Refers to Figure 2 A. Post-hoc paired comparisons of the CoP path length between trials in the four different sensory conditions. Significant differences are in bold type.*

|  | **EC** | | | | | | | |  | **EC-LT** | | | | | | | |
| --- | --- | --- | --- | --- | --- | --- | --- | --- | --- | --- | --- | --- | --- | --- | --- | --- | --- |
| Trial | 1 | 2 | 3 | 4 | 5 | 6 | 7 | 8 |  | 1 | 2 | 3 | 4 | 5 | 6 | 7 | 8 |
| 1 |  | **< 0.001** | **< 0.001** | **< 0.001** | **< 0.001** | **< 0.001** | **< 0.001** | **< 0.001** |  |  | **< 0.001** | **< 0.001** | **< 0.001** | **< 0.001** | **< 0.001** | **< 0.001** | **< 0.001** |
| 2 | **< 0.001** |  | **< 0.05** | **< 0.01** | **< 0.001** | **< 0.001** | **< 0.001** | **< 0.001** |  | **< 0.001** |  | 0.81 | 0.40 | 0.33 | **< 0.05** | 0.09 | **< 0.05** |
| 3 | **< 0.001** | **< 0.05** |  | 0.36 | **0.05** | 0.06 | 0.14 | **< 0.001** |  | **< 0.001** | 0.81 |  | 0.055 | 0.46 | 0.08 | 0.14 | **< 0.05** |
| 4 | **< 0.001** | **< 0.01** | 0.36 |  | 0.30 | 0.35 | 0.59 | **< 0.05** |  | **< 0.001** | 0.40 | 0.055 |  | 0.89 | 0.25 | 0.38 | 0.09 |
| 5 | **< 0.001** | **< 0.001** | **0.05** | 0.30 |  | 0.91 | 0.60 | 0.21 |  | **< 0.001** | 0.33 | 0.46 | 0.89 |  | 0.31 | 0.46 | 0.11 |
| 6 | **< 0.001** | **< 0.001** | 0.06 | 0.35 | 0.91 |  | 0.68 | 0.17 |  | **< 0.001** | **< 0.05** | 0.08 | 0.25 | 0.31 |  | 0.79 | 0.57 |
| 7 | **< 0.001** | **< 0.001** | 0.14 | 0.59 | 0.60 | 0.68 |  | 0.08 |  | **< 0.001** | 0.09 | 0.14 | 0.38 | 0.46 | 0.79 |  | 0.40 |
| 8 | **< 0.001** | **< 0.001** | **< 0.001** | **< 0.05** | 0.21 | 0.17 | 0.08 |  |  | **< 0.001** | **< 0.05** | **< 0.05** | 0.09 | 0.11 | 0.57 | 0.40 |  |
|  | | | | | | | | | | | | | | | | | |
|  | **EO** | | | | | | | |  | **EO-LT** | | | | | | | |
| Trial | 1 | 2 | 3 | 4 | 5 | 6 | 7 | 8 |  | 1 | 2 | 3 | 4 | 5 | 6 | 7 | 8 |
| 1 |  | 0.42 | 0.64 | 0.92 | 0.79 | 0.52 | 0.46 | 0.69 |  |  | 0.67 | 0.94 | 0.76 | 0.19 | 0.52 | 0.19 | 0.19 |
| 2 | 0.42 |  | 0.73 | 0.37 | 0.28 | 0.87 | 0.95 | 0.68 |  | 0.67 |  | 0.73 | 0.91 | 0.37 | 0.83 | 0.37 | 0.39 |
| 3 | 0.64 | 0.73 |  | 0.58 | 0.46 | 0.86 | 0.78 | 0.95 |  | 0.94 | 0.73 |  | 0.81 | 0.21 | 0.57 | 0.21 | 0.22 |
| 4 | 0.92 | 0.37 | 0.58 |  | 0.86 | 0.46 | 0.4 | 0.62 |  | 0.76 | 0.91 | 0.81 |  | 0.32 | 0.74 | 0.31 | 0.33 |
| 5 | 0.79 | 0.28 | 0.46 | 0.86 |  | 0.36 | 0.31 | 0.50 |  | 0.19 | 0.37 | 0.21 | 0.32 |  | 0.50 | 0.99 | 0.98 |
| 6 | 0.52 | 0.87 | 0.86 | 0.46 | 0.36 |  | 0.92 | 0.81 |  | 0.52 | 0.83 | 0.57 | 0.74 | 0.50 |  | 0.49 | 0.52 |
| 7 | 0.46 | 0.95 | 0.78 | 0.4 | 0.31 | 0.92 |  | 0.73 |  | 0.19 | 0.37 | 0.21 | 0.31 | 0.99 | 0.49 |  | 0.97 |
| 8 | 0.69 | 0.68 | 0.95 | 0.62 | 0.5 | 0.81 | 0.73 |  |  | 0.19 | 0.39 | 0.22 | 0.33 | 0.98 | 0.52 | 0.97 |  |
