# Supplementary material for: Balance Adaptation While Standing on a Compliant Base Depends on the Current Sensory Condition in Healthy Young Adults
Source: Front Hum Neurosci. 2022 Mar 25;16:839799. doi: 10.3389/fnhum.2022.839799 (PMC8989851; doi:10.3389/fnhum.2022.839799)
Supplement: Supplementary file 2 [file Table_2.DOCX]

***Table 2.*** *Refers to Figure 2 B. Post-hoc paired comparisons of the CoP sway area between trials in the four different sensory conditions. Significant differences are in bold type.*

|  | **EC** | | | | | | | |  | **EC-LT** | | | | | | | |
| --- | --- | --- | --- | --- | --- | --- | --- | --- | --- | --- | --- | --- | --- | --- | --- | --- | --- |
| Trial | 1 | 2 | 3 | 4 | 5 | 6 | 7 | 8 |  | 1 | 2 | 3 | 4 | 5 | 6 | 7 | 8 |
| 1 |  | 0.43 | 0.96 | 0.92 | 0.56 | 0.24 | 0.31 | 0.92 |  |  | 0.96 | 0.43 | 0.06 | 0.21 | 0.07 | **< 0.05** | **< 0.05** |
| 2 | 0.43 |  | 0.39 | 0.37 | 0.17 | **0.051** | 0.07 | 0.37 |  | 0.96 |  | 0.40 | 0.057 | 0.19 | 0.06 | **< 0.05** | **< 0.05** |
| 3 | 0.96 | 0.39 |  | 0.96 | 0.60 | 0.27 | 0.33 | 0.96 |  | 0.43 | 0.40 |  | 0.28 | 0.64 | 0.31 | 0.21 | 0.13 |
| 4 | 0.92 | 0.37 | 0.96 |  | 0.63 | 0.29 | 0.36 | 0.99 |  | 0.06 | 0.057 | 0.28 |  | 0.54 | 0.95 | 0.87 | 0.65 |
| 5 | 0.56 | 0.17 | 0.60 | 0.63 |  | 0.56 | 0.66 | 0.64 |  | 0.21 | 0.19 | 0.64 | 0.54 |  | 0.59 | 0.44 | 0.29 |
| 6 | 0.24 | 0.051 | 0.27 | 0.29 | 0.56 |  | 0.89 | 0.29 |  | 0.07 | 0.06 | 0.31 | 0.95 | 0.59 |  | 0.82 | 0.61 |
| 7 | 0.31 | 0.07 | 0.33 | 0.36 | 0.66 | 0.89 |  | 0.36 |  | **< 0.05** | **< 0.05** | 0.21 | 0.87 | 0.44 | 0.82 |  | 0.78 |
| 8 | 0.92 | 0.37 | 0.96 | 0.99 | 0.64 | 0.29 | 0.36 |  |  | **< 0.05** | **< 0.05** | 0.13 | 0.65 | 0.29 | 0.61 | 0.78 |  |
|  | | | | | | | | | | | | | | | | | |
|  | **EO** | | | | | | | |  | **EO-LT** | | | | | | | |
| Trial | 1 | 2 | 3 | 4 | 5 | 6 | 7 | 8 |  | 1 | 2 | 3 | 4 | 5 | 6 | 7 | 8 |
| 1 |  | 0.70 | 0.18 | 0.14 | **< 0.05** | **< 0.05** | **< 0.01** | 0.11 |  |  | 0.09 | 0.25 | 0.10 | **0.052** | **< 0.05** | **< 0.001** | **< 0.01** |
| 2 | 0.70 |  | 0.08 | 0.06 | **< 0.05** | **< 0.05** | **< 0.01** | **< 0.05** |  | 0.09 |  | 0.59 | 0.96 | 0.79 | 0.59 | 0.09 | 0.18 |
| 3 | 0.18 | 0.08 |  | 0.89 | 0.43 | 0.51 | 0.12 | 0.79 |  | 0.25 | 0.59 |  | 0.63 | 0.43 | 0.28 | **< 0.05** | 0.06 |
| 4 | 0.14 | 0.06 | 0.89 |  | 0.51 | 0.59 | 0.16 | 0.89 |  | 0.10 | 0.96 | 0.63 |  | 0.76 | 0.55 | 0.08 | 0.17 |
| 5 | **< 0.05** | **< 0.05** | 0.43 | 0.51 |  | 0.90 | 0.45 | 0.59 |  | **0.052** | 0.79 | 0.43 | 0.76 |  | 0.78 | 0.15 | 0.29 |
| 6 | **< 0.05** | **< 0.05** | 0.51 | 0.59 | 0.90 |  | 0.38 | 0.68 |  | **< 0.05** | 0.59 | 0.28 | 0.55 | 0.78 |  | 0.24 | 0.43 |
| 7 | **< 0.01** | **< 0.01** | 0.12 | 0.16 | 0.45 | 0.38 |  | 0.20 |  | **< 0.001** | 0.09 | **< 0.05** | 0.08 | 0.15 | 0.24 |  | 0.69 |
| 8 | 0.11 | **< 0.05** | 0.79 | 0.89 | 0.59 | 0.68 | 0.20 |  |  | **< 0.01** | 0.18 | 0.06 | 0.17 | 0.29 | 0.43 | 0.69 |  |
