# Supplementary material for: Balance Adaptation While Standing on a Compliant Base Depends on the Current Sensory Condition in Healthy Young Adults
Source: Front Hum Neurosci. 2022 Mar 25;16:839799. doi: 10.3389/fnhum.2022.839799 (PMC8989851; doi:10.3389/fnhum.2022.839799)
Supplement: Supplementary file 3 [file Table_3.DOCX]

***Table 3.*** *Refers to Figure 4 A. Post-hoc paired comparisons of the median frequency of the ML CoP spectrum between trials in the four different sensory conditions. Significant differences are in bold type.*

|  | **EC** | | | | | | | |  | **EC-LT** | | | | | | | |
| --- | --- | --- | --- | --- | --- | --- | --- | --- | --- | --- | --- | --- | --- | --- | --- | --- | --- |
| Trial | 1 | 2 | 3 | 4 | 5 | 6 | 7 | 8 |  | 1 | 2 | 3 | 4 | 5 | 6 | 7 | 8 |
| 1 |  | **< 0.01** | **< 0.001** | **< 0.001** | **< 0.001** | **< 0.001** | **< 0.001** | **< 0.001** |  |  | **< 0.001** | **< 0.001** | **< 0.001** | **< 0.001** | **< 0.001** | **< 0.001** | **< 0.001** |
| 2 | **< 0.01** |  | 0.08 | 0.09 | **< 0.01** | **< 0.01** | **< 0.05** | **< 0.001** |  | **< 0.001** |  | **< 0.05** | **< 0.01** | 0.06 | **< 0.001** | **< 0.001** | **< 0.001** |
| 3 | **< 0.001** | 0.08 |  | 0.94 | 0.13 | 0.18 | 0.63 | **< 0.01** |  | **< 0.001** | **< 0.05** |  | 0.27 | 0.89 | **< 0.05** | **< 0.05** | **< 0.01** |
| 4 | **< 0.001** | 0.09 | 0.94 |  | 0.12 | 0.16 | 0.58 | **< 0.01** |  | **< 0.001** | **< 0.01** | 0.27 |  | 0.22 | 0.24 | 0.27 | 0.08 |
| 5 | **< 0.001** | **< 0.01** | 0.13 | 0.12 |  | 0.87 | 0.31 | 0.26 |  | **< 0.001** | 0.06 | 0.89 | 0.22 |  | **< 0.05** | **< 0.05** | **< 0.01** |
| 6 | **< 0.001** | **< 0.01** | 0.18 | 0.16 | 0.87 |  | 0.39 | 0.19 |  | **< 0.001** | **< 0.001** | **< 0.05** | 0.24 | **< 0.05** |  | 0.94 | 0.58 |
| 7 | **< 0.001** | **< 0.05** | 0.63 | 0.58 | 0.31 | 0.39 |  | **< 0.05** |  | **< 0.001** | **< 0.001** | **< 0.05** | 0.27 | **< 0.05** | 0.94 |  | 0.53 |
| 8 | **< 0.001** | **< 0.001** | **< 0.01** | **< 0.01** | 0.26 | 0.19 | **< 0.05** |  |  | **< 0.001** | **< 0.001** | **< 0.01** | 0.08 | **< 0.01** | 0.58 | 0.53 |  |
|  | | | | | | | | | | | | | | | | | |
|  | **EO** | | | | | | | |  | **EO-LT** | | | | | | | |
| Trial | 1 | 2 | 3 | 4 | 5 | 6 | 7 | 8 |  | 1 | 2 | 3 | 4 | 5 | 6 | 7 | 8 |
| 1 |  | 0.48 | 0.18 | 0.50 | 0.61 | 0.12 | **< 0.05** | 0.09 |  |  | 0.16 | 0.83 | 0.33 | 0.98 | 0.89 | 0.71 | 0.44 |
| 2 | 0.48 |  | **< 0.05** | 0.17 | 0.23 | **< 0.05** | **< 0.01** | **< 0.05** |  | 0.16 |  | 0.23 | 0.66 | 0.16 | 0.20 | 0.29 | 0.52 |
| 3 | 0.18 | **< 0.05** |  | 0.50 | 0.40 | 0.82 | 0.29 | 0.73 |  | 0.83 | 0.23 |  | 0.44 | 0.85 | 0.94 | 0.87 | 0.58 |
| 4 | 0.50 | 0.17 | 0.50 |  | 0.87 | 0.36 | 0.08 | 0.31 |  | 0.33 | 0.66 | 0.44 |  | 0.34 | 0.41 | 0.55 | 0.83 |
| 5 | 0.61 | 0.23 | 0.40 | 0.87 |  | 0.28 | 0.06 | 0.24 |  | 0.98 | 0.16 | 0.85 | 0.34 |  | 0.91 | 0.73 | 0.45 |
| 6 | 0.12 | **< 0.05** | 0.82 | 0.36 | 0.28 |  | 0.40 | 0.91 |  | 0.89 | 0.20 | 0.94 | 0.41 | 0.91 |  | 0.82 | 0.53 |
| 7 | **< 0.05** | **< 0.01** | 0.29 | 0.08 | 0.06 | 0.40 |  | 0.47 |  | 0.71 | 0.29 | 0.87 | 0.55 | 0.73 | 0.82 |  | 0.69 |
| 8 | 0.09 | **< 0.05** | 0.73 | 0.31 | 0.24 | 0.91 | 0.47 |  |  | 0.44 | 0.52 | 0.58 | 0.83 | 0.45 | 0.53 | 0.69 |  |
