# Supplementary material for: Balance Adaptation While Standing on a Compliant Base Depends on the Current Sensory Condition in Healthy Young Adults
Source: Front Hum Neurosci. 2022 Mar 25;16:839799. doi: 10.3389/fnhum.2022.839799 (PMC8989851; doi:10.3389/fnhum.2022.839799)
Supplement: Supplementary file 4 [file Table_4.DOCX]

***Table 4.*** *Refers to Figure 4 B. Post-hoc paired comparisons of the median frequency of the AP CoP spectrum between trials in the four different sensory conditions. Significant differences are in bold type.*

|  | **EC** | | | | | | | |  | **EC-LT** | | | | | | | |
| --- | --- | --- | --- | --- | --- | --- | --- | --- | --- | --- | --- | --- | --- | --- | --- | --- | --- |
| Trial | 1 | 2 | 3 | 4 | 5 | 6 | 7 | 8 |  | 1 | 2 | 3 | 4 | 5 | 6 | 7 | 8 |
| 1 |  | **< 0.001** | **< 0.001** | **< 0.001** | **< 0.001** | **< 0.001** | **< 0.001** | **< 0.001** |  |  | **< 0.001** | **< 0.001** | **< 0.001** | **< 0.001** | **< 0.001** | **< 0.001** | **< 0.001** |
| 2 | **< 0.001** |  | 0.07 | **< 0.001** | **< 0.001** | **< 0.01** | **< 0.001** | **< 0.001** |  | **< 0.001** |  | 0.43 | **< 0.05** | **< 0.001** | **< 0.001** | **< 0.001** | **< 0.001** |
| 3 | **< 0.001** | 0.07 |  | 0.07 | **< 0.01** | 0.36 | 0.09 | 0.07 |  | **< 0.001** | 0.43 |  | 0.08 | **< 0.01** | **< 0.01** | **< 0.01** | **< 0.001** |
| 4 | **< 0.001** | **< 0.001** | 0.07 |  | 0.16 | 0.35 | 0.89 | 0.99 |  | **< 0.001** | **< 0.05** | 0.08 |  | 0.40 | 0.17 | 0.25 | 0.08 |
| 5 | **< 0.001** | **< 0.001** | **< 0.01** | 0.16 |  | **< 0.05** | 0.12 | 0.16 |  | **< 0.001** | **< 0.001** | **< 0.01** | 0.40 |  | 0.59 | 0.76 | 0.36 |
| 6 | **< 0.001** | **< 0.01** | 0.36 | 0.35 | **< 0.05** |  | 0.43 | 0.35 |  | **< 0.001** | **< 0.001** | **< 0.01** | 0.17 | 0.59 |  | 0.82 | 0.71 |
| 7 | **< 0.001** | **< 0.001** | 0.09 | 0.89 | 0.12 | 0.43 |  | 0.89 |  | **< 0.001** | **< 0.001** | **< 0.01** | 0.25 | 0.76 | 0.82 |  | 0.54 |
| 8 | **< 0.001** | **< 0.001** | 0.07 | 0.99 | 0.16 | 0.35 | 0.89 |  |  | **< 0.001** | **< 0.001** | **< 0.001** | 0.08 | 0.36 | 0.71 | 0.54 |  |
|  | | | | | | | | | | | | | | | | | |
|  | **EO** | | | | | | | |  | **EO-LT** | | | | | | | |
| Trial | 1 | 2 | 3 | 4 | 5 | 6 | 7 | 8 |  | 1 | 2 | 3 | 4 | 5 | 6 | 7 | 8 |
| 1 |  | 0.81 | 0.83 | 0.82 | 0.83 | 0.39 | 0.89 | 0.98 |  |  | 0.63 | 0.08 | 0.20 | 0.93 | 0.11 | **< 0.05** | **< 0.05** |
| 2 | 0.81 |  | 0.98 | 0.64 | 0.98 | 0.53 | 0.93 | 0.83 |  | 0.63 |  | 0.22 | 0.43 | 0.69 | 0.26 | 0.09 | 0.14 |
| 3 | 0.83 | 0.98 |  | 0.66 | 0.99 | 0.51 | 0.94 | 0.85 |  | 0.08 | 0.22 |  | 0.66 | 0.1 | 0.90 | 0.68 | 0.79 |
| 4 | 0.82 | 0.64 | 0.66 |  | 0.66 | 0.27 | 0.71 | 0.79 |  | 0.20 | 0.43 | 0.66 |  | 0.23 | 0.74 | 0.39 | 0.49 |
| 5 | 0.83 | 0.98 | 0.99 | 0.66 |  | 0.51 | 0.94 | 0.85 |  | 0.93 | 0.69 | 0.10 | 0.23 |  | 0.13 | **< 0.05** | 0.06 |
| 6 | 0.39 | 0.53 | 0.51 | 0.27 | 0.51 |  | 0.47 | 0.40 |  | 0.11 | 0.26 | 0.90 | 0.74 | 0.13 |  | 0.59 | 0.71 |
| 7 | 0.89 | 0.93 | 0.94 | 0.71 | 0.94 | 0.47 |  | 0.91 |  | **< 0.05** | 0.09 | 0.68 | 0.39 | **< 0.05** | 0.59 |  | 0.87 |
| 8 | 0.98 | 0.83 | 0.85 | 0.79 | 0.85 | 0.40 | 0.91 |  |  | **< 0.05** | 0.14 | 0.79 | 0.49 | 0.06 | 0.71 | 0.87 |  |
