# Supplementary material for: Balance Adaptation While Standing on a Compliant Base Depends on the Current Sensory Condition in Healthy Young Adults
Source: Front Hum Neurosci. 2022 Mar 25;16:839799. doi: 10.3389/fnhum.2022.839799 (PMC8989851; doi:10.3389/fnhum.2022.839799)
Supplement: Supplementary file 5 [file Table_5.DOCX]

***Table 5.*** *Refers to Figure 4 D. Post-hoc paired comparisons of the mean level of the ML CoP spectrum between trials in the four different sensory conditions. Significant differences are in bold type.*

|  | **EC** | | | | | | | |  | **EC-LT** | | | | | | | |
| --- | --- | --- | --- | --- | --- | --- | --- | --- | --- | --- | --- | --- | --- | --- | --- | --- | --- |
| Trial | 1 | 2 | 3 | 4 | 5 | 6 | 7 | 8 |  | 1 | 2 | 3 | 4 | 5 | 6 | 7 | 8 |
| 1 |  | 0.28 | 0.06 | **< 0.01** | **< 0.05** | 0.16 | **< 0.01** | **< 0.05** |  |  | 0.85 | 0.53 | 0.14 | 0.71 | 0.19 | 0.08 | 0.12 |
| 2 | 0.28 |  | 0.42 | **< 0.05** | 0.3 | 0.75 | 0.08 | 0.29 |  | 0.85 |  | 0.66 | 0.20 | 0.85 | 0.27 | 0.12 | 0.17 |
| 3 | 0.06 | 0.42 |  | 0.21 | 0.82 | 0.62 | 0.35 | 0.79 |  | 0.53 | 0.66 |  | 0.40 | 0.79 | 0.49 | 0.27 | 0.34 |
| 4 | **< 0.01** | **< 0.05** | 0.21 |  | 0.31 | 0.08 | 0.75 | 0.32 |  | 0.14 | 0.20 | 0.40 |  | 0.27 | 0.87 | 0.79 | 0.91 |
| 5 | **< 0.05** | 0.3 | 0.82 | 0.31 |  | 0.47 | 0.48 | 0.98 |  | 0.71 | 0.85 | 0.79 | 0.27 |  | 0.35 | 0.18 | 0.23 |
| 6 | 0.16 | 0.75 | 0.62 | 0.08 | 0.47 |  | 0.15 | 0.45 |  | 0.19 | 0.27 | 0.49 | 0.87 | .35 |  | 0.67 | 0.78 |
| 7 | **< 0.01** | 0.08 | 0.35 | 0.75 | 0.48 | 0.15 |  | 0.50 |  | 0.08 | 0.12 | 0.27 | 0.79 | 0.18 | 0.67 |  | 0.88 |
| 8 | **< 0.05** | 0.29 | 0.79 | 0.32 | 0.98 | 0.45 | 0.50 |  |  | 0.12 | 0.17 | 0.34 | 0.91 | 0.23 | 0.78 | 0.88 |  |
|  | | | | | | | | | | | | | | | | | |
|  | **EO** | | | | | | | |  | **EO-LT** | | | | | | | |
| Trial | 1 | 2 | 3 | 4 | 5 | 6 | 7 | 8 |  | 1 | 2 | 3 | 4 | 5 | 6 | 7 | 8 |
| 1 |  | 0.65 | 0.58 | 0.20 | 0.31 | 0.49 | 0.13 | 0.46 |  |  | 0.13 | 0.61 | 0.36 | 0.15 | 0.38 | **< 0.05** | 0.25 |
| 2 | 0.65 |  | 0.31 | 0.08 | 0.14 | 0.25 | **0.05** | 0.23 |  | 0.13 |  | 0.31 | 0.55 | 0.95 | 0.53 | 0.50 | 0.73 |
| 3 | 0.58 | 0.31 |  | 0.47 | 0.65 | 0.89 | 0.34 | 0.86 |  | 0.61 | 0.31 |  | 0.68 | 0.34 | 0.71 | 0.09 | 0.51 |
| 4 | 0.20 | 0.08 | 0.47 |  | 0.79 | 0.56 | 0.81 | 0.59 |  | 0.36 | 0.55 | 0.68 |  | 0.59 | 0.97 | 0.20 | 0.81 |
| 5 | 0.31 | 0.14 | 0.65 | 0.79 |  | 0.74 | 0.62 | 0.78 |  | 0.15 | 0.95 | 0.34 | 0.59 |  | 0.56 | 0.46 | 0.77 |
| 6 | 0.49 | 0.25 | 0.89 | 0.56 | 0.74 |  | 0.41 | 0.96 |  | 0.38 | 0.53 | 0.71 | 0.97 | 0.56 |  | 0.19 | 0.77 |
| 7 | 0.13 | **0.05** | 0.34 | 0.81 | 0.62 | 0.41 |  | 0.44 |  | **< 0.05** | 0.50 | 0.09 | 0.20 | 0.46 | 0.19 |  | 0.31 |
| 8 | 0.46 | 0.23 | 0.86 | 0.59 | 0.78 | 0.96 | 0.44 |  |  | 0.25 | 0.73 | 0.51 | 0.81 | 0.77 | 0.77 | 0.31 |  |
