# Supplementary material for: Balance Adaptation While Standing on a Compliant Base Depends on the Current Sensory Condition in Healthy Young Adults
Source: Front Hum Neurosci. 2022 Mar 25;16:839799. doi: 10.3389/fnhum.2022.839799 (PMC8989851; doi:10.3389/fnhum.2022.839799)
Supplement: Supplementary file 6 [file Table_6.DOCX]

***Table 6.*** *Refers to Figure 4 E. Post-hoc paired comparisons of the mean level of the AP CoP spectrum between trials in the four different sensory conditions. Significant differences are in bold type.*

|  | **EC** | | | | | | | |  | **EC-LT** | | | | | | | |
| --- | --- | --- | --- | --- | --- | --- | --- | --- | --- | --- | --- | --- | --- | --- | --- | --- | --- |
| Trial | 1 | 2 | 3 | 4 | 5 | 6 | 7 | 8 |  | 1 | 2 | 3 | 4 | 5 | 6 | 7 | 8 |
| 1 |  | 0.76 | **< 0.05** | **< 0.001** | **< 0.001** | **< 0.001** | **< 0.001** | **< 0.05** |  |  | 0.97 | 0.63 | 0.12 | 0.27 | 0.16 | 0.10 | 0.09 |
| 2 | 0.76 |  | 0.09 | **< 0.01** | **< 0.01** | **< 0.01** | **< 0.001** | **< 0.05** |  | 0.97 |  | 0.61 | 0.12 | 0.26 | 0.15 | 0.09 | 0.08 |
| 3 | **< 0.05** | 0.09 |  | 0.18 | 0.14 | 0.11 | **< 0.05** | 0.59 |  | 0.63 | 0.61 |  | 0.29 | 0.54 | 0.36 | 0.25 | 0.22 |
| 4 | **< 0.001** | **< 0.01** | 0.18 |  | 0.91 | 0.80 | 0.50 | 0.42 |  | 0.12 | 0.12 | 0.29 |  | 0.66 | 0.9 | 0.93 | 0.85 |
| 5 | **< 0.001** | **< 0.01** | 0.14 | 0.91 |  | 0.89 | 0.58 | 0.36 |  | 0.27 | 0.26 | 0.54 | 0.66 |  | 0.75 | 0.59 | 0.53 |
| 6 | **< 0.001** | **< 0.01** | 0.11 | 0.80 | 0.89 |  | 0.68 | 0.29 |  | 0.16 | 0.15 | 0.36 | 0.9 | 0.75 |  | 0.82 | 0.75 |
| 7 | **< 0.001** | **< 0.001** | **< 0.05** | 0.50 | 0.58 | 0.68 |  | 0.14 |  | 0.10 | 0.09 | 0.25 | 0.93 | 0.59 | 0.82 |  | 0.93 |
| 8 | **< 0.05** | **< 0.05** | 0.59 | 0.42 | 0.36 | 0.29 | 0.14 |  |  | 0.09 | 0.08 | 0.22 | 0.85 | 0.53 | 0.75 | 0.93 |  |
|  | | | | | | | | | | | | | | | | | |
|  | **EO** | | | | | | | |  | **EO-LT** | | | | | | | |
| Trial | 1 | 2 | 3 | 4 | 5 | 6 | 7 | 8 |  | 1 | 2 | 3 | 4 | 5 | 6 | 7 | 8 |
| 1 |  | 0.93 | 0.12 | 0.43 | 0.07 | **< 0.05** | **< 0.05** | **< 0.05** |  |  | 0.30 | 0.29 | 0.21 | 0.19 | **< 0.05** | **< 0.01** | **< 0.01** |
| 2 | 0.93 |  | 0.14 | 0.48 | 0.08 | **< 0.05** | **< 0.05** | **0.054** |  | 0.30 |  | 0.98 | 0.81 | 0.76 | 0.32 | 0.09 | **0.052** |
| 3 | 0.12 | 0.14 |  | 0.44 | 0.79 | 0.53 | 0.33 | 0.65 |  | 0.29 | 0.98 |  | 0.83 | 0.78 | 0.33 | 0.09 | 0.06 |
| 4 | 0.43 | 0.48 | 0.44 |  | 0.30 | 0.16 | 0.08 | 0.22 |  | 0.21 | 0.81 | 0.83 |  | 0.95 | 0.45 | 0.15 | 0.09 |
| 5 | 0.07 | 0.08 | 0.79 | 0.30 |  | 0.71 | 0.48 | 0.85 |  | 0.19 | 0.76 | 0.78 | 0.95 |  | 0.49 | 0.17 | 0.1 |
| 6 | **< 0.05** | **< 0.05** | 0.53 | 0.16 | 0.71 |  | 0.74 | 0.86 |  | **< 0.05** | 0.32 | 0.33 | 0.45 | 0.49 |  | 0.49 | 0.34 |
| 7 | **< 0.05** | **< 0.05** | 0.33 | 0.08 | 0.48 | 0.74 |  | 0.61 |  | **< 0.01** | 0.09 | 0.09 | 0.15 | 0.17 | 0.49 |  | 0.79 |
| 8 | **< 0.05** | **0.054** | 0.65 | 0.22 | 0.85 | 0.86 | 0.61 |  |  | **< 0.01** | **0.052** | 0.06 | 0.09 | 0.1 | 0.34 | 0.79 |  |
