# Supplementary material for: Balance Adaptation While Standing on a Compliant Base Depends on the Current Sensory Condition in Healthy Young Adults
Source: Front Hum Neurosci. 2022 Mar 25;16:839799. doi: 10.3389/fnhum.2022.839799 (PMC8989851; doi:10.3389/fnhum.2022.839799)
Supplement: Supplementary file 7 [file Table_7.DOCX]

|  |  | **ML** | | | |  | **AP** | | | |
| --- | --- | --- | --- | --- | --- | --- | --- | --- | --- | --- |
| **W** | **Condition** | EC | EC-LT | EO | EO-LT |  | EC | EC-LT | EO | EO-LT |
| 1 | EC |  | **< 0.01** | 0.25 | **< 0.05** |  |  | **< 0.001** | 0.05 | 0.23 |
|  | EC-LT | **< 0.01** |  | 0.13 | **< 0.001** |  | **< 0.001** |  | **< 0.001** | **< 0.05** |
|  | EO | 0.25 | 0.12 |  | **< 0.001** |  | 0.05 | **< 0.001** |  | **< 0.01** |
|  | EO-LT | **< 0.05** | **< 0.001** | **< 0.001** |  |  | 0.23 | **< 0.05** | **< 0.01** |  |
| 2 | EC |  | **< 0.05** | 0.24 | 0.35 |  |  | **< 0.001** | 0.06 | 0.36 |
|  | EC-LT | **< 0.05** |  | **< 0.001** | **< 0.001** |  | **< 0.001** |  | **< 0.001** | **< 0.01** |
|  | EO | 0.24 | **< 0.001** |  | 0.80 |  | 0.06 | **< 0.001** |  | **< 0.01** |
|  | EO-LT | 0.35 | **< 0.001** | 0.80 |  |  | 0.36 | **< 0.01** | **< 0.01** |  |
| 3 | EC |  | 0.36 | **< 0.05** | **< 0.05** |  |  | **< 0.01** | **< 0.01** | **< 0.001** |
|  | EC-LT | 0.36 |  | 0.17 | 0.10 |  | **< 0.01** |  | 0.49 | 0.89 |
|  | EO | **< 0.05** | 0.17 |  | 0.78 |  | **< 0.01** | 0.49 |  | 0.42 |
|  | EO-LT | **< 0.05** | 0.10 | 0.78 |  |  | **< 0.001** | 0.89 | 0.42 |  |
| 4 | EC |  | 0.49 | **< 0.01** | **< 0.001** |  |  | 0.74 | **< 0.001** | **< 0.001** |
|  | EC-LT | 0.49 |  | **< 0.05** | **< 0.001** |  | 0.74 |  | **< 0.01** | **< 0.001** |
|  | EO | **< 0.01** | **< 0.05** |  | **< 0.01** |  | **< 0.001** | **< 0.01** |  | **< 0.05** |
|  | EO-LT | **< 0.001** | **< 0.001** | **< 0.01** |  |  | **< 0.001** | **< 0.001** | **< 0.05** |  |
| 5 | EC |  | 0.58 | 0.14 | 0.27 |  |  | **< 0.05** | 0.58 | **< 0.001** |
|  | EC-LT | 0.58 |  | 0.35 | 0.09 |  | **< 0.05** |  | **< 0.05** | **< 0.001** |
|  | EO | 0.14 | 0.35 |  | **< 0.05** |  | 0.58 | **< 0.05** |  | **< 0.001** |
|  | EO-LT | 0.27 | 0.09 | **< 0.05** |  |  | **< 0.001** | **< 0.001** | **< 0.001** |  |
| 6 | EC |  | 0.26 | 0.46 | **< 0.01** |  |  | **< 0.01** | 0.90 | **< 0.05** |
|  | EC-LT | 0.26 |  | 0.69 | 0.07 |  | **< 0.01** |  | **< 0.01** | **< 0.001** |
|  | EO | 0.46 | 0.69 |  | **< 0.05** |  | 0.90 | **< 0.01** |  | **< 0.05** |
|  | EO-LT | **< 0.01** | 0.07 | **< 0.05** |  |  | **< 0.05** | **< 0.001** | **< 0.05** |  |

***Table 7.****Refers to Figure 6. Post-hoc paired comparisons between sensory conditions of the slopes of the regression lines calculated on the mean level of the ML and AP CoP spectra in the six frequency windows. Significant differences are in bold type.*
